# Characterizing the circular RNA landscape in phloem sap of *Brassica napus*

**DOI:** 10.1371/journal.pone.0347473

**Published:** 2026-04-29

**Authors:** Kim Lara Lühmann, Zsófia Fekete, Rita Fernandes, Julia Kehr

**Affiliations:** 1 Department of Biology, University of Hamburg, Institute of Plant Science and Microbiology, Molecular Plant Genetics, Hamburg, Germany; 2 Department of Environmental and Biological Sciences, Faculty of Science, University of Eastern Finland, Forestry and Technology, Joensuu, Finland; 3 Hungarian University of Agriculture and Life Science, Genetics and Biotechnology Institute, Gödöllõ, Hungary; Shandong Agricultural University, CHINA

## Abstract

In plants, long-distance transport and phloem-mediated signal distribution play crucial roles in regulating stress adaptation and development. Phloem sap contains various types of RNAs, including small RNAs (sRNAs), messenger RNAs (mRNAs), and long noncoding RNAs (lncRNAs). Recently, endogenous circular RNAs have been identified in phloem sap of apple trees. Some phloem RNAs have been shown to have long-distance signaling functions, but for most, no functions have yet been determined. Due to their stability, circRNAs are interesting candidates with potential functions in long-distance signaling. Therefore, we aimed to characterize the circRNA content in the phloem sap of the crop plant *Brassica napus*. To achieve this, we performed Illumina sequencing of rRNA-depleted, circRNA-enriched, and sRNA libraries. The analysis revealed 1,734 distinct circRNAs in the phloem sap of *B. napus*. Of these, we validated ten circRNAs by PCR amplification and Sanger sequencing of their back-splicing junctions (BSJs). Using circ-Panel Nanopore sequencing, we investigated the full-length sequences of 14 circRNAs from phloem sap and leaf samples, identifying seven high-confidence candidates that exhibit potential intron retention and isoform variation across the two tissues. The investigation of potential interaction partners from phloem circRNA identified miRNA target sites on multiple circRNAs, particularly for known phloem-mobile miRNAs like miR156, miR169, and miR395. With Microscale Thermophoresis (MST), we were able to show the ability of the abundant RNA-binding protein BnGRP7 to bind the phloem circRNA circBnaANL2(7,8) with a dissociation constant of around 1 µM, raising questions about the involvement of RBPs in circRNA transport, stabilization, and function in phloem sap.

## Introduction

To respond to the diverse conditions that plants experience throughout their lifetimes, they require an efficient internal communication system. This communication is facilitated by the long-distance transport of nutrients and macromolecules through the vascular tissue. As part of the vascular tissue, the phloem not only transports sugars from photosynthetic tissues to roots but also mediates long-distance transport of other molecules like phytohormones [1], proteins [2–4], and RNAs [5–9] in response to different stimuli. The sieve elements (SE) of the phloem lack a nucleus and functional ribosomes, thus proteins and RNAs have to be imported from adjacent companion cells (CCs) into SEs [10–12]. Until now, the selection of RNAs for long-distance transport is not well understood. RNA types identified in phloem samples range from small RNAs (sRNAs) [5,13–16] to transfer RNAs (tRNAs) and tRNA halves [10] to messenger RNAs (mRNAs) [7,17,18] and ribosomal RNAs (rRNAs) [10]. Thus, the phloem contains a broad RNA landscape. Evidence that RNAs can transmit information is available for only a few RNAs, mostly sRNAs. Under nutrient deficiency, for example, specific microRNAs (miRNAs) can be transported from shoots to roots to regulate nutrient allocation [5,19–21]. A few examples describe mRNAs as mobile regulators of development, including *BEL5*, which is involved in tuber formation regulation [22], and *GAI* and *GAIP*, which influence leaf shape [23]. While most studies have focused on sRNAs and mRNAs, the diversity of RNA species in phloem sap suggests that less explored RNA classes may also contribute to long-distance signaling.

Recently, circular RNAs (circRNAs) have been found in phloem sap of *Malus domestica* (*M. domestica*). Over the past decade, the discovery of endogenous circRNAs in mammals [24], Drosophila [25], rats and mice [26,27], Archaea [28], and plants [29,30] has raised questions about their function. CircRNAs are long, non-coding, single-stranded RNAs with a covalent link between their 5’ and 3’ ends [24]. This link, also called a back-splicing junction (BSJ), is formed by alternative splicing that can be regulated by splicing factors and complementary sequences [25–27]. Due to their circular nature, circRNAs are considered to be more stable than linear RNAs, as they cannot be degraded by exonucleases [24]. Because of their proposed higher stability, circRNAs represent interesting candidates for long-distance transport and signaling functions. This idea is supported by the ability of plant pathogens like viroids, which are single-stranded, non-coding circularized RNAs with a size of around 300 nucleotides (nt), to use the phloem to systemically infect the plant [28,29]. They are not protected by coat proteins or lipids. Instead, their stability is granted by their circular genome, preventing degradation by exonucleases [24]. Thus, they might utilize a system that was originally developed for endogenous circRNAs.

CircRNAs have been identified across many plant species like *Arabidopsis thaliana* (*A.thaliana*), *Oryza sativa* (*O. sativa*), *Solanum lycopersicum* (*S. lycopersicum*), *Hordeum vulgare* (*H. vulgare*), and *M. domestica* [30–34]. Generally, circRNAs are known to possess various functions, including miRNA sponging and regulation [35,36], protein sponging [37], and transcriptional and translational regulation [38]. The expression of circRNAs varies depending on the tissue [34], stresses such as drought [39], salt [40], heat [41], or cold [42], or the developmental stage [43] of the plant. Therefore, they are assumed to have regulatory functions in stress response and development, like other long non-coding RNAs and miRNAs, which makes them interesting candidates for potential long-distance signaling during stress response.

Despite the emerging roles of circRNAs in stress response, little is known about their possible functions in long-distance transport and signaling. They are a highly understudied RNA class in phloem sap. Therefore, our study aimed to reveal the circRNA content of the phloem and analyze their potential interaction partners. We choose *Brassica napus* (*B. napus*) as a model plant, because it is a globally important crop species, is closely related to *A. thaliana*, and established protocols for phloem sap sampling and RNA analysis exist [5,13,44]. Moreover, circRNAs have already been identified in *B. napus* leaves [45]. To achieve our goal, we employed Illumina sequencing of rRNA-depleted and circRNA-enriched libraries to obtain a comprehensive overview of circRNAs present in phloem sap, which resulted in a list of 1,734 circRNAs. The identified circRNAs were further validated and analyzed for their potential functions using in silico analysis, Nanopore long-read sequencing for isoform discovery, and Microscale Thermophoresis to study protein-RNA interactions. Notably, we were able to predict potential circRNA-miRNA interactions and demonstrate that an abundant phloem RNA-binding protein, BnGRP7, is capable of binding a circRNA, suggesting a potential mechanism for circRNA transport or function in the phloem.

## Results

### The phloem sap of *B. napus* contains circRNAs

By now, RNAs from phloem sap have predominantly been sequenced using poly (A)- enriched or small RNA libraries, thereby missing out on RNAs that do not contain a poly(A)-tail or are longer than 50 nucleotides. Recent studies have demonstrated that lncRNAs and circRNAs are present in the phloem sap of *M. domestica* (apple tree) [34,46]. To increase our knowledge of the circRNA content in the phloem of the relevant crop species oilseed rape, we performed rRNA-depleted and circRNA-enriched library preparation and sequencing at Novogene (Cambridge, UK). For rRNA-depleted libraries, we used three biological replicates from phloem RNA samples. Two biological replicates were used for circRNA-enriched libraries of phloem sap RNAs and leaf RNAs, respectively. Phred30 scores were above 90% for all replicates and all libraries (S1a and S1b Tables in S2 File), and the error rate distribution along reads was between 0.02–0.04% (S1b and S2b Tables in S2 File).

**Fig 1:**
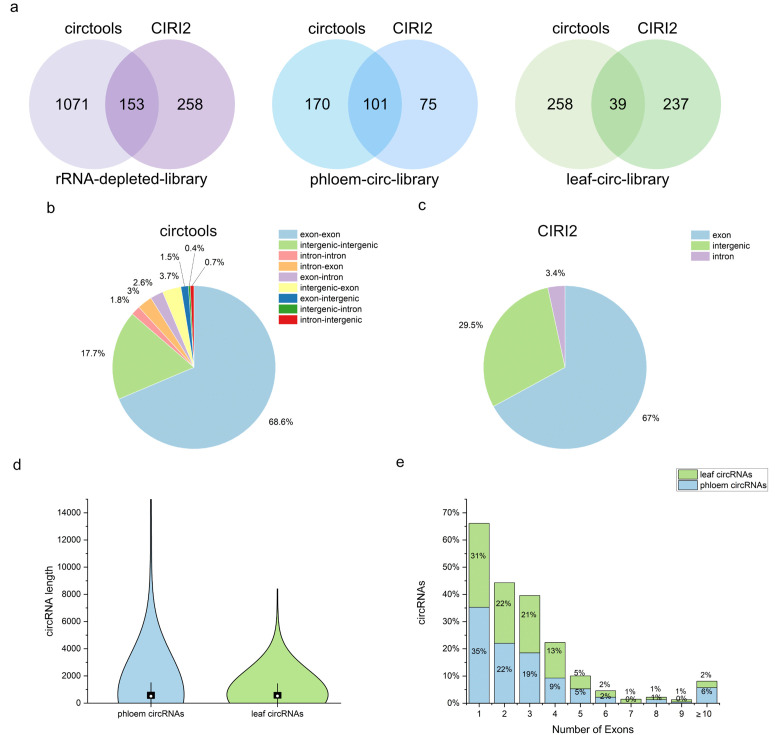
Content of circRNAs in total phloem and leaf RNA detected with circtools and CIRI2. The circRNA content in phloem samples was detected in both lnc- and circRNA-enriched libraries, whereas circRNAs in the leaf samples were only identified in the circRNA-enriched sequencing data. **(a)** Venn diagrams showing the overlap of circRNAs detected by circtools and CIRI2 in phloem lnc-RNA data (left), as well as in phloem (middle) and leaf (right) circRNA-enriched samples. **(b)-(c)** Pie-charts with mapping regions of phloem circRNAs by circtools (b) and CIRI2 (c) in circRNA-enriched samples. **(d)** Violin plot representing the length distribution of circRNAs in phloem sap (blue) and leaf samples (green). The length was calculated using the distance between the BSJ. The x-axis displays the length in nucleotides. The white point displays the median length (507 nt for phloem circRNAs, 525 nt for leaf circRNAs). Significance was calculated using the Mann-Whitney test. No significant difference was found (p = 0.79). **(e)** The stacked bar graph displays the distribution of circRNAs with certain numbers of exons in phloem (blue) and leaf (green) samples. The x-axis displays the number of exons, while the y-axis displays the percentage of circRNAs.

Since all circRNA prediction tools are based on different algorithms and utilize the alignments of different aligners (e.g., RNA-STAR and BWA-mem2), not every circRNA is detected by each tool [47,48]. Therefore, we used Circtools (DCC) and CIRI2 to predict circRNAs in phloem sap (rRNA-depleted and circRNA-enriched libraries) and leaves (circRNA-enriched libraries), as this combination has been described as a robust approach for circRNA detection [47]. The full bioinformatic workflow can be found in an overview (Fig S10 in S3 File), and publications and GitHub repositories for both tools are mentioned in the methods. In the rRNA-depleted library, we identified 1,224 potential circRNAs with circtools, each supported by at least one BSJ read in a single replicate. In parallel, CIRI2 detected 411 circRNAs, each with a minimum of two BSJ reads in one replicate. Among those circRNAs, 153 were detected by both tools (Fig 1a, S2 and S3 Tables in S2 File). In the circRNA-enriched libraries of phloem sap RNA (phloem-circ-library), 170 and 75 circRNAs were detected using circtools and CIRI2, respectively. A total of 101 circRNAs were identified by both tools (Fig 1a, S4 and S5 Tables in S2 File). Comparing the detected circRNAs from rRNA-depleted and circRNA-enriched libraries from phloem sap, 1432 circRNAs were identified in the rRNA-depleted library only, while 51 circRNAs were detected in both the rRNA-depleted and circRNA-enriched libraries (S3b Fig in S3 File). In leaf samples (leaf-circ-library), circtools identified 258 and CIRI2 237 unique circRNAs, with an overlap of 39 circRNAs (Fig 1a, S6 and S7 Table in S2 File). Comparing circRNAs from leaf-circ-library and phloem-circ-library, a total of 109 circRNAs were exclusively detected in the phloem, whereas 242 circRNAs were present in both phloem and leaf (S3a Fig in S3 File). Differential expression analysis between circRNAs in phloem sap and leaf samples has not been performed since the low read counts per circRNA and only two biological replicates would not result in a robust and reliable result [49].

The majority of circRNAs in phloem were derived from known exons, with 65–70% in the phloem circRNA-library (Fig 1 b,c), while nearly 30% of the circRNAs were derived partially or completely from intergenic regions (Fig 1 b,c). For circRNAs identified with circtools from rRNA-depleted library sequencing of phloem, similar findings were observed, but CIRI2 detected a considerably larger portion of circRNAs derived from intergenic regions, with 45% and only 50% derived from known exons (S3d Fig in S3 File). In leaves, the distribution of the annotation of circRNAs was similar, with a slightly higher tendency toward exon-derived circRNAs, which might be noticeable (S3c Fig in S3 File). The distribution of circRNA length in phloem samples was broader than the distribution in leaf samples, but the length of circRNAs showed no significant difference (p = 0.79) in phloem and leaf samples (Fig 1d). The median length of phloem circRNAs was 508 nt, and the median length of leaf circRNAs was 525 nt. Most circRNAs found in phloem samples were composed of one exon from their cognate mRNA (35%), and many covered two (22%) to three exons (19%) (Fig 1e). Similarly, most leaf circRNAs (31%) consisted of a single exon and showed an exon distribution similar to phloem RNAs (Fig 1e).

### Validation of circRNA candidates

**Fig 2:**
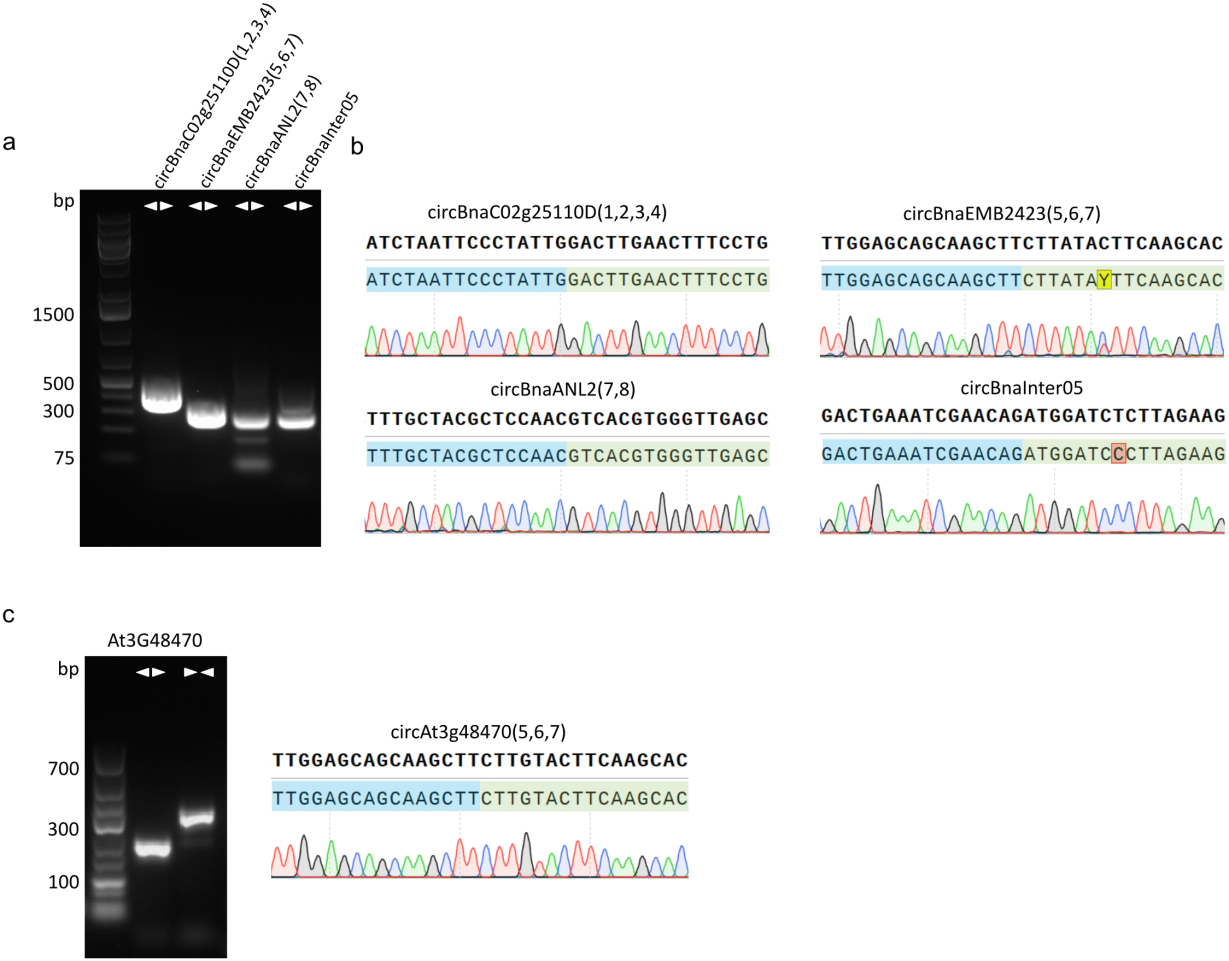
BSJ amplification and sequencing with divergent primer pairs. (**a)** Agarose gel (1.5%) with amplicons of BSJ-PCRs targeting four different circRNAs: circRNABnaC02g25110D, circBnaEMB2423(5,6,7), circBnaANL2(7,8) and circBnaInter05. **(b)** Sanger sequencing results of BSJ amplicons of circBnaC02g25110D(1,2,3,4), circBnaEMB2423(5,6,7), circBnaANL2(7,8), circBnaInter05 and circAt3g48470(5,6,7). **(c)** Agarose gel (1.5%) with amplicons of At3g48470 with divergent (first lane) and convergent (second lane) primer pairs as well as Sanger sequencing results of BSJ amplicon of circAt3g48470(5,6,7).

The BSJs of 22 circRNAs were validated via RT-PCR (Fig 2a, S5 Fig in S3 File), and ten amplicons were sequenced for further validation of the BSJ sequence (Fig 2b, S6 Fig in S3 File). The circRNA circBnaEMB2423(5,6,7) showed high similarities to a circRNA derived from *A. thaliana* (At3g48470, PlantcircBase ID: ath_circ_026560), which is predicted to contain exons 5–7. Using PCR with a divergent primer pair and amplicon sequencing, the BSJ between exon 7 and exon 5 of circAt3g48470 (5,6,7) was confirmed (Fig 2c). The genomic location and the read count of all circRNAs mentioned with circRNA-IDs can be found in Supplementary Table 8 in S2 File.

### Long read sequencing of circRNAs

**Fig 3:**
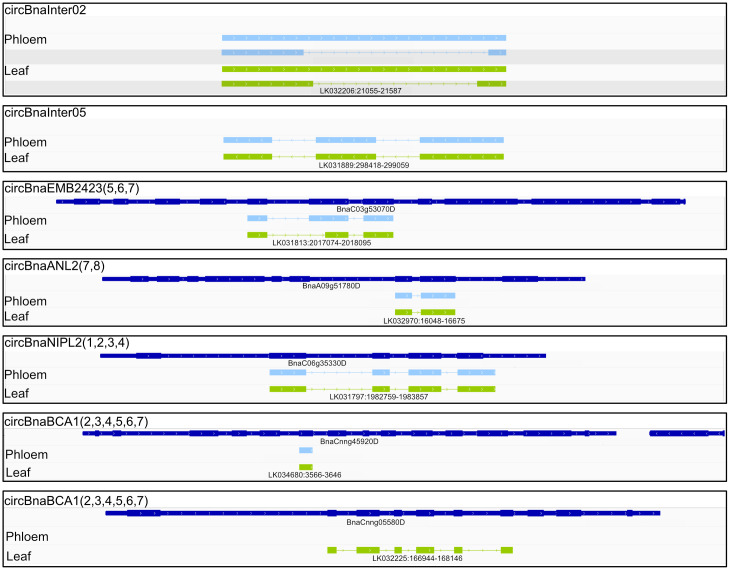
Isoforms of six circRNAs identified by CIRI-long from phloem and leaf samples. The gene annotation is shown in dark blue. Isoforms from phloem samples are displayed in light blue and isoforms from leaf samples are displayed in green. No gene annotations are shown for the intergenic circRNAs. Visualized using IGV (v2.11.9) [52,53].

To investigate the full-length sequences of the detected circRNAs, we employed circRNA-Panel sequencing [50] of two biological replicates from leaf and phloem samples. We obtained full-length sequences of 14 circRNAs. Of these, twelve were amplified from both phloem and leaf samples, and two from leaf samples only. Nanopore sequencing data were analyzed using CIRI-Long [51]. For seven circRNAs, the same BSJs as previously identified with Illumina sequencing were identified by CIRI-long (Figs 3; S9c, S9d, S9l-S9v Tables in S2 File). Together with their validation through PCR (all seven) and BSJ sequencing (all except circBnaNIPL2(1,2,3,4)), these circRNAs resemble our high-confidence candidates. For six other circRNAs, circRNA isoforms with BSJs at the same locus close to the original sequence were found (S9a, S9b, S9e-S9k Tables in S2 File), and for one, no isoform from the same locus was found (S9w and S9x Table in S2 File). Most circRNAs with previously identified BSJs had a single isoform sharing the same BSJ. Only circBnaInter02 (in phloem and leaf) had two isoforms per BSJ (Fig 3). For the seven high-confidence circRNAs, only circBnaEMB2423(5,6,7) showed a difference between phloem and leaf isoforms (Fig 3). Here, only half of exon 6 was present in the isoform retrieved from leaf samples.

### GO-term and KEGG-pathway enrichments of phloem circRNAs

**Fig 4:**
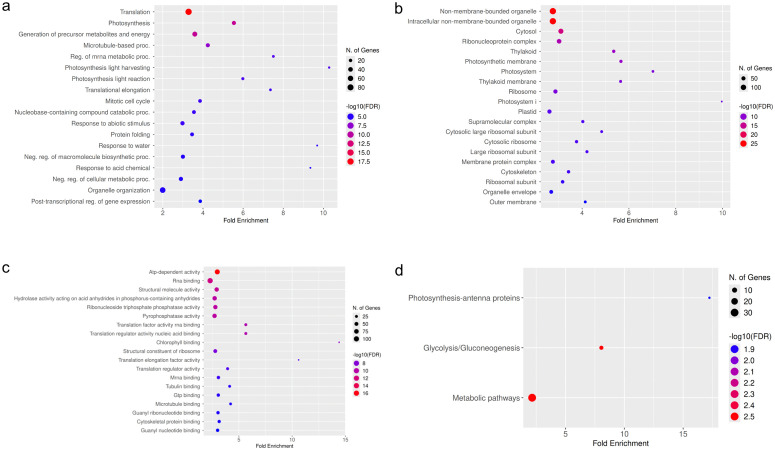
Gene ontology (GO) and KEGG-pathway analysis of genes hosting circRNAs. Analysis was performed using ShinyGO v0.85 [54] with all B. napus genes as background. **(a)** GO-terms for Biological Process **(b)** GO-terms for Cellular Component **(c)** GO-terms for Molecular Function **(d)** KEGG-pathway analysis.

To obtain an initial overview of the possible functions of circRNAs in the phloem, we performed GO term and KEGG analyses using circRNA parent gene annotations with all *B. napus* genes as background. In biological process, genes of different regulatory processes like the negative regulation macromolecule biosynthetic and cellular metabolic processes, regulation of mRNA metabolic processes, translation and the posttranscriptional regulation of gene expression were enriched (Fig 4a, S10 Table in S2 File). In cellular components, among others, genes in the category cytosol and ribonucleoprotein complex were enriched (Fig 4b, S11 Table in S2 File). Furthermore, genes with the molecular functions of RNA binding, binding of other molecules and in translational functions were enriched (Fig 4c, S12 Table in S2 File). The KEGG-pathway analysis showed the enrichment of genes in metabolic pathways and glycolysis (Fig 4d, S13 Table in S2 File).

### Phloem circRNAs contain potential miRNA target sites

Interest in circRNAs began to rise about a decade ago. During this period, several functions of circRNAs were elucidated. One example is the regulatory function of Os-circANK on miRNA 398b in plants, and the miRNA sponging of CDR1as circRNA in humans, which has over 70 seed-target sites for miR-7. As there are several miRNAs present in phloem sap that show systemic movement and signaling functions, it is possible that circRNAs interact with these miRNAs. Through these interactions, circRNAs could support the transport of miRNAs, protect them, or regulate their functions. To investigate if phloem circRNAs could have such potential functions within the phloem, we applied Illumina sequencing to gain data on the sRNA content of *B. napus* phloem sap.

**Fig 5:**
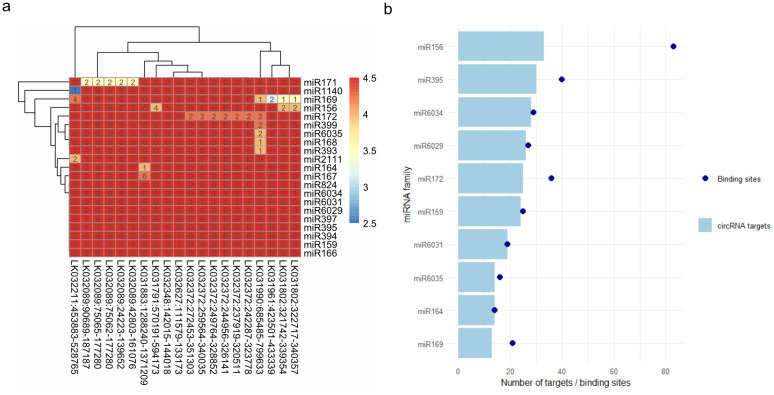
miRNA target sites on circRNAs. **(a)** Heatmap displaying the number of miRNA target sites per circRNA. 20 circRNAs with the highest number of binding sites were selected. **(b)** Number of circRNA targets and total binding sites from ten miRNA families with the highest number of binding sites.

The bioinformatics analysis by Novogene resulted in a list of mature miRNAs (S15 Table in S2 File). MiRNAs that did not match known miRNAs in miRBase were assigned the prefix novel_ and a number. We employed TargetFinder [55] and psRNA Target [56, 57,58] to identify miRNA target sites in all phloem circRNAs. Thereby, we wanted to obtain initial insights into possible circRNA – miRNA interactions. Fewer miRNA targets were detected with TargetFinder than with psRNA Target (S16 and S17 Table in S2 File). The results of both tools were combined, and 20 circRNAs with the highest number of miRNA target sites were selected. To act as a miRNA sponge, the circRNA is expected to have multiple miRNA seed sequence target sites. The phloem miRNAs in our set showed multiple binding sites on some circRNAs, but most had few binding sites and high scores (colors in the heatmap), suggesting weaker binding (Fig 5a). For example, miR156 has multiple binding sites on multiple circRNAs, but most scores were high and clustered on circRNAs that seem to be overlapping isoforms (Fig 5a). Similarly, miR172 had multiple targets on the same circRNAs but also had scores of 4. Other miRNAs, such as miR171 and miR169, showed fewer binding sites but lower scores, indicating a greater likelihood of true miRNA targets (Fig 5a, S16 and S17 Table in S2 File). The miRNA family miR156, with more than 80 reported binding sites, had the most binding sites on phloem circRNAs, targeting more than 10 distinct circRNAs. Other miRNAs, such as miR169, miR171, miR172, miR395, and miR6034, also had 10 or more predicted binding sites (Fig 5b). All miRNAs have been reported in phloem sap in previous studies [5,13,21].

### The abundant phloem RBP is capable of binding circRNA

Not only are circRNAs known to interact with miRNAs, but they were also shown to interact with RBPs. Therefore, we asked whether known phloem RBPs, such as BnGRP7, can bind circRNAs. To test this, we used in vitro transcribed and circularized circBnaANL2(7,8). Employing MST, the binding affinity of BnGRP7 towards circBnaANL2(7,8) was determined. The K_d_ was 1.27 µM with a standard deviation (SD) of 0.12 µM (Fig 5b), which is similar compared to the binding affinities of BnGRP7 to other RNAs [59].

## Discussion

The phloem serves as an important long-distance signaling route for regulatory RNAs, including several miRNAs and selected mRNAs [19,20,22,23]. These mobile RNAs play critical roles in coordinating plant development and stress responses across distant tissues. While numerous RNA species contribute to stress adaptation, their potential role in systemic signaling remains largely unexplored. Recently, circRNAs have gained increased attention as regulatory RNAs involved in stress adaptation and development. Some have been shown to regulate gene expression by acting as miRNA sponges [35]. Their defining characteristic is their circular structure, which provides enhanced stability and resistance to exonuclease-mediated degradation [24]. Even though circRNAs have been identified in plants and linked to stress responses, their possible role in long-distance signaling is still unclear. Due to their structural stability and regulatory potential, circRNAs are promising candidates for systemic signaling molecules within the phloem. Therefore, elucidating the circRNA content and their potential functions in phloem sap could provide new insights in RNA-based regulation and signaling. Here, we demonstrate the presence of circRNAs in the phloem sap of *B. napus* utilizing rRNA-depleted and circRNA-enriched sequencing data and further validate the BSJ of circRNAs. *In silico* analysis identified potential miRNA targets and binding sites on circRNAs. MST experiments highlighted interactions between phloem circRNAs and the abundant phloem protein BnGRP7, suggesting roles in protection, transport, or regulation.

### Conservation of circRNA features across species

We performed circRNA identification with circtools [60,61] and CIRI2 [62]. Both tools identified circRNAs in rRNA-depleted sequencing libraries and in circRNA-enriched sequencing libraries, but only a few were identified by both tools (Fig 1a). The majority of circRNAs have been identified by only one of the tools. Most likely, the discrepancy between circtools and CIRI2 detected circRNAs is caused by the use of different mapping tools before BSJ identification and the different approaches that the programs use to identify BSJ reads as circRNAs [61,62]. Both programs have a high identification accuracy but differ in sensitivity for detecting certain circRNAs [47].

Since some circRNAs are susceptible to RNase R treatment, they cannot be detected in circRNA-enriched libraries [35], which could explain the small overlap of only 51 circRNAs comparing the circRNAs detected in rRNA-depleted and the circRNA-enriched library (S3a Fig in S2 File). Furthermore, some circRNAs are expressed at such low levels that they are detectable only in circRNA-enriched libraries. Most likely, both factors influence the small overlap between the rRNA-depleted and circRNA-enriched libraries. It is therefore important to combine both library strategies to gain a more comprehensive overview of all circRNAs. The overlap of circRNAs detected in phloem and leaf samples was with 80% noticeably higher (S3a Fig in S2 File). In *M. domestica*, only 27% of circRNAs overlapped between phloem and leaf samples [34]. The observed difference could be due to the fact that *B. napus* and *M. domestica* are different species and not closely related. An additional reason might be that circRNAs in *M. domestica* were identified only with find_circ [34], which has not been used in our study. Despite these differences, the results of both studies suggest that there are tissue-specific circRNAs, which is further supported by the identification of tissue-specific circRNAs in mammals [63].

Compared to previous studies, the distribution of circRNAs between exonic, intronic, and intergenic in *B. napus* phloem and leaf samples (Fig 1b,c) was similar to other plants. In *A. thaliana,* around 80% of circRNAs are derived from exons. In phloem sap of *M. domestica*, over 65% of the circRNAs were exonic, and the smallest portion (5%) were derived from intronic regions [34]. A previous study in *B. napus* identified circRNAs in leaves and the amount of intronic circRNAs was similar to our findings, but a smaller number of exonic circRNAs, accounting for around 50%, was detected [45]. Among the circRNAs identified in our study, 14 were previously reported in *B. napus* [45]. Two of these circRNAs, circBnaBnaANL2(7,8) and circBnaEMB2423(5,6,7), could be validated in this study (Fig 2a,b).

CircRNAs in phloem and leaf samples had similar length distributions (Fig 1d). Most circRNAs had a size around 200–600 nt, which is similar to the length distribution of circRNAs identified in recent studies of *B. napus*, *B. rapa* and *M. domestica* [34,45,64]. The number of exons in the identified circRNAs was similar between phloem and leaf. In both tissues, most circRNAs contained one exon (Fig 1e). In the shoot apical meristem of soybean, most circRNAs contained 2–3 exons [65] while in *B. rapa* pollen, most circRNAs contained one exon [43], thus there might be differences in circRNA structure between species or tissues.

Taken together, circRNAs identified in this study showed similar features compared to circRNAs identified in previous studies. This supports the reliability of the methods chosen. Furthermore, circRNA length and origin seem to be conserved between tissues and plant species.

### Validation of phloem circRNAs

Even though prediction tools like circtools and CIRI2 are quite accurate, some of the identified circRNAs can be false positives [47]. For validation, we performed RT-PCR with divergent primers to confirm the BSJs. We validated 22 circRNAs (Fig 3, S5 Fig in S2 File) by PCR. Further validation of the BSJ sequence was achieved for nine circRNAs, including circBnaANL2(7,8), circBnaBCA1(1,2,3,4,5,6,7), and circBnaEMB2423(5,6,7) by Sanger sequencing (Fig 3, S6 Fig in S2 File). In a previous study on circRNAs in *B. napus*, 14 circRNAs have been validated using RT-qPCR. However, no validation via Sanger sequencing of the BSJ amplicons has been performed [45]. CircRNAs were also identified and validated in other species. For example, in *O. sativa*, ten exonic circRNAs were validated, while no intergenic, intronic or other categorized circRNAs could be validated [30].

The identification of circRNAs using short read sequencing data relies on reads spanning the BSJ, as reads mapping to RNA sequences between the BSJ may also originate from the linear parent RNA and the complete sequences cannot be determined. Additionally, circRNAs can undergo splicing. Thus, introns as well as exons can be removed, while micro exons can be retained [66,50]. Since most studies investigated the full-length sequences of circRNAs computationally, our study represents an important first step to identify full-length sequences of plant circRNAs, their intron retention, and their reliability. A few studies with circRNA full-length sequencing data exist from *O. sativa* [67], *Phyllostachys edulis* (*P. edulis*) [68] and *Lotus japonicus* (*L. japonicus*) [69]. The study on *L. japonicus* circRNAs utilized circPanel-LRS to characterize circRNA sequences. Consistent with our results, they identified validated circRNAs, such as circCCR4(4,5), along with additional isoforms from the same gene locus, with the same or other BSJ [69]. We identified seven out of 14 circRNAs with isoforms utilizing the same BSJ as detected with circtools and CIRI2, in addition to multiple other isoforms with different BSJs (Fig 3). For the other seven circRNAs, only isoforms with different BSJ but from the same locus were identified. Among these were circBnaInter01 and circBnaC02g25110D(1,2,3,4), for which we have validated the BSJ by PCR and Sanger sequencing. The other circRNAs with BSJ discrepancies between Illumina and Nanopore sequencing have not been validated with Sanger sequencing. In rice, complex splice sites were significantly more frequent at splice sites that produce circRNAs compared to linear RNAs, resulting in more than two donor and acceptor sites [70]. In combination with specific amplification and sequencing of circRNAs, this could lead to the enrichment and detection of circRNA isoforms with slightly different BSJs from the same locus. Additionally, Nanopore sequencing is still associated with higher base-calling error rates than Illumina sequencing, despite the improvements over the past few years [71]. These errors can lead to wrong alignments, which could cause the detection of false BSJ. In addition, not all circRNA detection tools use the same algorithm and aligner outputs, which can result in the identification of slightly different BSJs [47]. Therefore, CIRI-long may have identified slightly different BSJ compared to circtools and CIRI2 because of variation in the algorithm. Overall, this demonstrates that the identification with multiple tools and the validation of BSJs is crucial to identify true circRNAs.

### Potential functions of phloem circRNAs

Many studies on plant circRNA use a GO-term and KEGG-enrichment analysis based on their annotated parent genes to gain first insights in which processes the identified circRNAs might be involved in [40,72]. In *A. thaliana*, parent genes of circRNAs showed enrichments in multiple GO-terms and pathways similar to our findings (Fig 4, S10–S13 Table in S2 File). For example, genes in regulatory processes, metabolic processes and with the molecular function “binding” were upregulated [73]. Similarly, in the KEGG-analysis genes in pathways like biosynthesis of amino acids, mRNA surveillance, and ribosomes were upregulated [73]. A similar result was described for *M. domestica*. The enrichment of circRNA parent genes of different gene clusters was investigated, and it was shown that the enriched clusters were similar across all tissues [34]. The GO-term and KEGG enrichment we performed on phloem circRNAs (with all *B. napus* genes as background) resulted in similar enriched GO-terms and KEGG-pathways (Fig 4) as the ones detected for *A. thaliana* and *M. domestica*. This comparison suggests that circRNAs are derived from genes with similar functions across species and tissues under normal growth conditions.

One of the first reported functions of circRNAs was the ability of CDR1as to act as a miRNA sponge for miR-7, with more than 70 target sites for the seed region of miR-7 [35]. This function enables circRNAs to bind miRNAs so that they cannot interact with their target RNAs. Similar miRNA-binding functions were described for the circRNA Os-circANK, which can regulate miR398b in plants [36]. In phloem sap, circRNAs could regulate miRNAs by hindering their binding to their main target, or they could transport miRNAs through the phloem and stabilize them. We identified multiple miRNA targets in phloem circRNAs (Fig 5a), some of which have multiple binding sites on the same circRNA (Fig 5b). Among the miRNAs with multiple binding sites, we found some well-studied RNAs such as miR156, miR159, miR164, miR169, miR172, and miR395 (Fig 5b). Some of them have been reported to move systemically through the phloem and exhibit signaling functions, such as miR395 and miR169 [5,13,21]. These miRNAs could be bound by phloem circRNAs, which may stabilize them during phloem transport or prevent them from reaching their destination. Thus, circRNAs might buffer miRNAs until they are needed for an immediate stress response. Another interesting observation has been made in soybean. Here, two circRNAs had binding sites for miR156 or miR172, both of which are involved in the developmental phase transition to flowering [65]. It has been suggested that circ-SEC5A, which is upregulated under short-day conditions, may reduce the availability of miR156 through its four binding sites. Consequently, this could upregulate flowering transition genes such as SPL9 [65]. CircRNAs in phloem sap that contain miR156 and miR172 binding sites may exhibit similar functions and regulate developmental processes. In *B. napus* leaves, three circRNAs that were differentially expressed during herbicide treatment had 55, 24, and five miRNA binding sites, respectively [73]. Similarly, differentially expressed circRNAs with potential miRNA-sponging functions have been identified in stress responses of other plant species [36,45]. It would be interesting to examine whether the circRNA content of the phloem changes under different stresses and whether stress-specific circRNAs have more miRNA targets with lower scores, indicating a more reliable interaction. Many phloem-mobile miRNAs play important roles in stress responses, and phloem circRNAs with binding sites might regulate them. These functions could be tested in the future using CRISPR/Cas9 to create knock-out mutants of specific circRNAs as recently demonstrated in rice [74]. In summary, phloem circRNAs could interact with phloem-mobile miRNAs, potentially influencing the movement, availability, or stability of miRNAs. This interaction could establish an additional layer of regulation for long-distance signaling and stress response.

In addition to their miRNA-sponging function, circRNAs have been reported to regulate gene expression. For example, the circRNA derived from exon 6 of SEPALLATA3 can bind to its own DNA locus and form an R-loop [38]. This R-loop leads to the formation of an alternatively spliced isoform of the linear transcript, which influences flower development [38]. Phloem circRNAs could have similar functions and might be transported through the phloem to their destination, where they could influence alternative splicing in response to stress or developmental changes.

**Fig 6:**
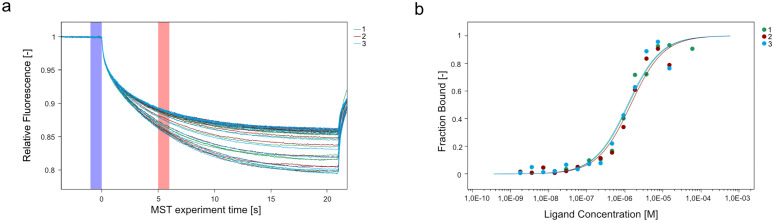
Microscale thermophoresis (MST) of BnGRP7 with circBnaANL2(7,8). **(a)** MST-traces of three independent measurements of BnGRP7 with circBnaANL2(7,8). The starting point of the thermophoresis is at 0 s, and the relative fluorescence was measured over the course of the time. The blue bar represents the MST-off fluorescence ΔF_0_ and the red bar represents the timepoint (5-6 **s)** ΔF1, which was used to calculate the fraction bound curves. **(b)** The ligand concentration plotted against the fraction bound percentage of circBnaANL2(7,8) bound to BnGRP7 for three independent measurements.

Phloem RBPs have been shown to interact with RNAs that travel through the phloem, including the phloem RBP CsPP2, which facilitates the transport of Hop stunt viroids [75]. In addition, phloem RBPs seem to show little RNA binding selectivity, as recent studies with PARCL and GRP7 described [59,76], which suggests that other RBPs in phloem sap may also interact with and transport circRNAs. One intriguing candidate is AthGRP7, which has been found in apoplastic fluid alongside AGO2 and circRNAs [77]. To determine if GRP7 can bind phloem circRNAs, we conducted MST measurements with BnGRP7 and the circRNA circBnaANL2(7,8). The results indicated an interaction with a Kd of 1.27 µM ± 0.12 µM (Fig 6b), highlighting the potential of GRP7 to interact with circRNAs in the phloem. Furthermore, GRP7 binds to extracellular RNAs of various sizes, ranging from 50 to 500 nt, which may include circRNAs [77]. A mutation in GRP7 resulted in a reduced amount of circRNAs in apoplastic fluid, leading to the hypothesis that GRP7 may play a role in circRNA secretion or stabilization of circRNAs [77]. Combined with our results, this suggests that GRP7 interacts with circRNAs; however, the extent of this interaction and its ultimate function remain to be elucidated. Overall, our results and previous studies indicate that endogenous circRNAs could interact with RBPs, which could enable the transport of circRNAs through the phloem or into the apoplast, or provide additional protection against degradation.

## Conclusions

In this study, we identified a total of 1734 circRNAs in phloem sap of *B. napus*. Of these, 22 circRNAs were experimentally validated through RT-PCR, and 10 circRNAs were additionally validated by Sanger sequencing of the BSJ. Using circPanel-seq, we further explored the full-length sequences of 14 circRNAs, yielding 7 high-confidence candidates. This suggests that validating each circRNA is crucial for confirming their true BSJ and structure. Notably, we found that multiple circRNAs contain target sequences for known phloem miRNAs, such as miR156 and miR169. These miRNA-circRNA interactions may serve various functions, including the regulation and stabilization of miRNAs, as well as their transport through the phloem. Additionally, we demonstrated that the known phloem protein GRP7 can bind to circRNAs, indicating that such interactions may play a role in circRNA stabilization, regulation, or localization. Collectively, our findings provide evidence that circRNAs present in phloem sap may participate in regulatory processes and long-distance signaling. Further studies are needed to elucidate the precise functions of circRNAs and their interactions with proteins and small RNAs in the phloem.

## Methods

### Plant growth

*B. napus* cv. Drakkar were grown on soil-sand mixture (3:1, soil was the Einheitserde (Einheitserdewerk, Uetersen, DE) in the green house at 18 °C, light intensity of 170 µmol/m^2^/s and a light/dark cycle of 16 h/8 h. Phloem sap and leaves were harvested after eight weeks of growth.

*A. thaliana* (Col-0) were grown on soil-sand-expanded clay mixture (65%/25%/10%) in a climate chamber at 22 °C, with a light intensity of 100 µmol/ m^2^/s and a light/dark cycle of 16 h/8 h. Leaves were harvested after three weeks of growth. All seeds were produced in house.

### Phloem sap and leaf sampling

Phloem sap was collected as described previously [44]. In short, plants shortly before flowering (around 8 weeks old) were watered and consecutively punctured multiple times at the inflorescence stem at noon (12 pm). The first drops were removed with a tissue to avoid contaminating cell debris, and the subsequently exuding phloem sap drops were collected (every 5 minutes for an hour) into cooled 1.5 ml reaction tubes. The sap of up to 5 plants was pooled to around 250 µl, resembling one biological replicate, frozen in liquid nitrogen, and stored at −80 °C until further use.

For leaf material, young leaves of 8-week-old plants were cut, directly frozen in liquid nitrogen, and stored at −80 °C until further use.

For circRNA-enriched sequencing and Nanopore long-read sequencing, phloem samples originated from the same plants as the leaf material. Leaf material was collected directly after phloem sampling, frozen in liquid nitrogen, and stored at −80 °C.

### RNA isolation and reverse transcription

For one biological replicate for PCR validation, circRNA-enriched libraries, and Nanopore sequencing, one leaf from each of five different plants was combined and ground in liquid nitrogen. For phloem sap, one biological replicate consisted of combined phloem sap from five different plants.

100 mg of ground leaf material and 250 µl phloem sap were used for each RNA isolation.

The RNA isolation was performed with TRIzol^TM^ for leaf material and TRIzol^TM^ LS for phloem sap, both in combination with an RNA Clean & Concentrator-25 RNA-Kit (Zymo Research) according to the manufacturer’s protocol. The TRIzol^TM^ protocol was followed until the aqueous phase was separated. Consequently, the manufacturer’s instructions for the RNA Clean & Cocentrator-25 RNA-Kit were followed, and DNaseI digestion was performed on the column according to the manufacturer’s protocol.

For reverse transcription of RNA to cDNA, Maxima H- Reverse transcriptase was used according to the manufacturer’s instructions with an input of 300−500 ng/µl RNA. 100 U/µl Maxima H- was used in each reaction. Following reverse transcription, 1 µl RNaseH was added, and the reaction was incubated for 20 min at 37 °C and 20 min at 80 °C. For later use, cDNA was stored at −20 °C.

### Illumina Sequencing

For rRNA-depleted library preparation and sequencing, three biological replicates (each comprising one leaf from a single plant) were generated. For circRNA-enriched sequencing library preparation and sequencing, two biological replicates for leaf samples (each replicate consisted of one leaf from five different plants) and two biological replicates for phloem samples (each replicate consisted of 50 µl phloem sap from five different plants) were prepared.

RNA Samples for Illumina Sequencing were assessed for quality using Bioanalyzer and NanoDrop measurements (S9a,b Fig in S2 File). Phloem RNA was assessed for purity by performing PCR targeting the small RuBisCO subunit and Thioredoxin h (S8c Fig in S2 File). Subsequently, the RNA was sent for library preparation (small RNA library, rRNA-depleted library, and circRNA-enriched library) and Illumina sequencing to Novogene (Cambridge, UK). For all three libraries the standard service was selected.

Briefly, for small RNA library preparation, the NEBNext® Multiplex Small RNA Library Prep Set for Illumina® (NEB, USA) was used according to the manufacturer’s instructions. For Illumina sequencing, the samples were clustered on a cBot Cluster Generation System using the TruSeq SR Cluster Kit v3-cBot-HS (Illumina) following the manufacturer’s instructions and sequenced on an Illumina platform, generating 50 bp reads.

For rRNA-depleted library preparation and sequencing (lncRNA sequencing by Novogene), RNA was treated with the Ribo-Zero kit for rRNA depletion, followed by fragmentation with the fragmentation buffer. Reverse transcription was performed with random hexamers primers, and second-strand synthesis was initiated by adding a second-strand synthesis buffer, dNTPs, RNaseH, and DNA polymerase I. After terminal repair, A-ligation followed by sequencing adaptor ligation were performed. Size selection and PCR completed the cDNA library.

For circRNA library preparation and sequencing, Novogene performed rRNA removal with 2 µg of total RNA. Following, the RNA was treated with 3 U RNaseR (Epicentre, USA) per 1 µg RNA to remove residual linear RNA. From the remaining RNA, libraries were prepared using the NEBNext® Ultra™ Directional RNA Library Prep Kit for Illumina® (NEB, USA). Size selection of fragments between 250–300 bp was achieved by purification of the library fragments with AMPure XP system (Beckman Coulter, USA). 3 µl USER Enzyme (NEB, USA) was incubated at 37 °C for 15 min and at 95 °C for 5 min prior to PCR. PCR amplification of the library was performed using Phusion High-Fidelity Polymerase, universal primers, and an index (X) primer.

### Nanopore sequencing

To identify full-length sequences and isoforms of circRNAs, Nanopore Panel sequencing was performed as previously described, with some modifications [50]. In short, two biological replicates of phloem and leaf samples were used for RNA isolation. Biological replicates from leaves consisted of one leaf from each of five plants, which were combined before grinding and RNA isolation. Biological replicates of phloem sap consisted of 50 µl of phloem sap from five plants (totaling 250 µl), which were combined prior to RNA isolation.

RNase R digestion was performed using 1 U RNase R per 1 µg of total RNA at 37 °C for 30 min. Following reverse transcription, whole circRNAs were amplified using circRNA-specific divergent primers that lay back-to-back. Amplicons were purified using Monarch® PCR&DNA Cleanup Kit (NEB). For library preparation, the Nanopore barcoding kit SQK-NBD114.96 was used to add a barcode to each amplicon and to anneal the adapters. Therefore, the protocol v14-sqk-nbd114–96-NBA_9170_v114_revK_15Sep2022-minion from Nanopore was followed. Two sequencing libraries were prepared and sequenced on the same flow cell, sequentially. The flow cell was cleaned between the runs using the flow cell wash kit EXP-WSH004.

Base calling of sequencing data was performed using MinKNOW v23.07.15 (Nanopore) with high-accuracy base calling and barcode trimming.

### Bioinformatic analysis of sequencing data of the small RNA library

The bioinformatic analysis of sequencing data from the small RNA libray was performed by Novogene. Briefly, for the analysis of small RNA libraries reads were mapped with Bowtie v0.12.9 [78,79] (main parameters: -v 0 -k 1) against the *Brassica napus* genome assembly AST_PRJEB5043_v1 release-42 from Ensembl and analyzed using miREvo v1.1 [80] (main parameter: -i -r -M -m -k -p 10 -g 50000), mirdeep2 v0.0.5 [81] and ViennaRNA v2.1.1 [82,83] (integrated tool, main parameter: quantifier.pl -p -m -r -y -g 0 -T 10 default). Repeats were analyzed using RepeatMasker v4.0.3 (main parameter: -species –nolow -no_is –norna –pa 8).

### Bioinformatic analysis of sequencing data for circRNA detection

To detect circRNAs in sequencing data of *B. napus* total phloem RNA and total leaf RNA, Illumina sequencing reads from the lnc-library (only phloem) and circRNA-enriched library, two different prediction programs were used, circtools [60] and CIRI2 [62]. A flowchart depicting the bioinformatic workflow can be found in the supplementary file S3 Fig. S10 in S3 File.

For prediction with circtools v1.2.2 (GitHub https://github.com/dieterich-lab/circtools) [60], the reads were mapped against the *Brassica napus* genome assembly AST_PRJEB5043_v1 release-59 from Ensembl with STAR v2.5.2b-2 [84] on Galaxy Europe Server with these changes to the standard settings: sjdbOverhang: 149, quantMode: GeneCounts, outSAMattributes: all, outFilterMultimapNmax: 20, outFilterMismatchNmax: 999, outFilterMismatchNoverLmax 0.05, outFilterscoreMin: 1, outFilterMatchNminOverLread: 0.7, alignIntronMin: 20, alignIntronMax: 1,000,000, alignMatesGapMax: 1,000,000, alignSJoverhangMin: 15, alignSJDBoverhangMin: 10, twopassMode: Basic, chimSegmentMin: 15, chimScoreMin: 15, chimJunctionOverhangMin: 15. Circtools Detect module (DCC) was used to identification circRNA candidates with standard settings without -M and -R flags and with -Nr 0 2 (or Nr 0 3 for lnc-Data) [60,61].

For circRNA identification with CIRI2 v2.0.6 (https://sourceforge.net/projects/ciri/files/CIRI2/) [62], the reads were mapped against *Brassica napus* genome assembly AST_PRJEB5043_v1 release-59 from Ensembl with BWA-MEM2 v2.2.1 [85] using the standard settings. Following, CIRI2 was used to detect BSJs from SAM-files generated by BWA-MEM2. For CIRI2, standard settings were used with -low as stringency filter. Tables of biological replicates were merged using Access2019 (Office 2019).

### GO- and KEGG-analysis

To investigate which GO-terms and KEGG-pathways were enriched in circRNAs, ShinyGO v0.85 [54] (accessed 26.09.2025) was used with standard settings (FDR cutoff: 0.05, pathway size: min. 2) and *Brassica napus* as selected species. The gene background for GO and KEGG analysis included all *B. napus* genes.

### miRNA binding site analysis

The miRNA binding sites of circRNAs were investigated using TargetFinder (v1 on Galaxy Europe) [55] and psRNA Target (v2 2017 release) [56, 57,58]. The list of phloem miRNAs was based on Illumina sequencing data from the sRNA library and on bioinformatic analysis by Novogene, as described in the dedicated section of the materials and methods. The lists of phloem circRNAs from rRNA-depleted and circRNA-enriched library sequencing and the detection by circtools and CIRI2 were combined, and duplicates were removed. To identify miRNA target sites, TargetFinder was used with the standard settings (prediction score cut-off: 4) and psRNA Target with the settings schema V2 20217 without allowing bulge (Expectation: 5, penalty for GU-pair: 0.5, penalty for other mismatches: 1, extra weight in seed region: 1, seed region: 2–13 nt, number of allowed mismatches in seed region: 2, HSP size: 19, translation inhibition range: 10–11 nt).

### CircRNA identification and isoform prediction with CIRI-Long and Minimap2-mapping

To identify and investigate the isoforms of the circRNAs, CIRI-Long v1.1.0 (GitHub https://github.com/bioinfo-biols/CIRI-long) [51] was used with default settings. As input, the basecalled and barcode trimmed reads of each barcode from Nanopore Panel sequencing were combined into one file and the reads for each barcode were submitted for circRNA identification with CIRI-Long and the genome assembly AST_PRJEB5043_v1 release-59 of *Brassica napus* from Ensembl plants. Following this, isoform collapse (merging of similar predicted isoforms into one) and output visualization were run with standard settings, with output data of CIRI-Long circRNA identification. Bed files were visualized with IGV genome browser v2.11.9 [52]. For further analysis of read distribution from each barcode, fasta files generated by CIRI-Long were aligned to the genome assembly AST_PRJEB5043_v1 release-59 of *Brassica napus* from Ensembl plants using Minimap2 v2.28 + galaxy0 [86] on the Galaxy Europe server using the Oxford Nanopore read to reference mapping profile of preset options. A list of barcode IDs and primers used for each circRNA can be found in S9 Table in S2 File.

### Validation of back splicing junctions

To validate the back-splicing junctions of circRNA candidates, PCR was performed using cDNA (see RNA isolation and reverse transcription) and divergent primer pairs. Therefore, 0.5−1 µl DreamTaq Polymerase, 5 µl DreamTaq buffer, 2 µl cDNA, 2 µl 10 mM forward primer, 2 µl 10 mM reverse primer, and 2 µl 10 mM dNTPs were used in a total PCR volume of 50 µl. Following PCR, the whole PCR reaction was loaded onto a 1.5−2% agarose gel and run for 45 min at 120 V. Agarose gels were imaged and amplicons were cut out of the gel and purified with the Monarch® Gel Extraction Kit (NEB). Amplicons were either send directly for Sanger sequencing at Microsynth Seqlab (Göttingen, Germany) or cloned into pUC57. For cloning into pUC57, amplicons were introduced by simultaneously cutting pUC57 with SmaI and ligation with T4 ligase at 16 °C for 16 h. 5 µl of this reaction were transformed into *E. coli* by incubating it with 100 µl competent XL10 on ice for 15 min, heat shock at 42 °C for 45 s and regeneration in 200 µl LB for 30 min-1 h at 37 °C. Bacteria were plated on LB agarose plates containing 100 µg/ml Carbenicillin. Colonies were tested with standard colony PCR using M13 F and R primers with an annealing temperature of 55 °C and 30 cycles. Overnight cultures of positive colonies were grown in 5 ml LB and 100 µg/ml Carbenicillin and plasmids were isolated using the NucleoSpin Plasmid easy pure kit (Macherey&Nagel, Düren, Germany) and send for Sanger sequencing at Microsynth Seqlab (Göttingen, Germany) with the M13 F primer.

PCR validation of the BSJs was done with at least two biological replicates. Sanger sequencing of PCR amplicons was performed for one sample.

### RNA *in vitro* transcription and *in vitro* circularization

For *in vitro* transcription and circularization, the full-length sequence of circBnaANL2(7,8) was cloned into a plasmid between the 5’- and 3’ intron halves of the PIE T4 td group 1 intron (Supplementary Fig. S8 in S3 File) described by Rausch et al. 2021 [87]. RNA was *in vitro* transcribed as described before [59]. In short, the template for *in vitro* transcription was generated by amplifying the DNA sequence with a forward primer harboring the T7 polymerase promoter. T7-RNA Polymerase from Thermo Fisher Scientific was used according to the manufacturer’s protocol and the reaction was incubated for 2 h at 37 °C. Following this, 10 U DNase I was added to the reaction and incubated 30 min at 37 °C. The reaction was stopped with 50 mM EDTA, and the RNA was purified using the RNA Clean & Concentrator-25 RNA-Kit (Zymo Research) according to manufactures protocol. Subsequently, *in vitro* circularization was performed by incubating the RNA for 15 min at 55 °C with 2 mM GTP. For linear RNA removal, RNase R digestion was performed for 30 min at 37 °C with 1 U RNase R per 1 µg RNA. Subsequently, RNA was purified using the RNA Clean & Conentrator-25 RNA-Kit (Zymo Research). To test if the RNA was circular, reverse transcription with random hexamer primers was performed and the generated cDNA was used to amplify the newly created BSJ with a divergent primer pair (S9 Fig in S3 File).

### Microscale Thermophoresis

MST measurements were performed as previously described [59]. In short, a 16-step titration with a starting concentration of 110 µM protein (BnGRP7) was set up for each measurement (Buffer: 25 mM Tris-HCl pH 8.0, 150 mM NaCl, 1 mM DTT and 0.1 mg/ml BSA). 40 nM of cy3-labeled RNA was added resulting in 20 nM finale concentration. The samples were incubated for 2 min. All 16 samples were loaded into Monolith NT.115 Standard treated capillaries (Nanotemper, Germany) and placed onto the loading rack. The first measurement started with standard settings and fluorescence autodetect. The measurements for the replicates were performed at the fluorescence percentage picked by the autodetect mode of the first measurement. Three independent measurements were performed and analyzed separately with MO.Affinity Analysis software v2.1.2. Mean and standard deviation were calculated using OriginPro v2024.

## Supporting information

S1 Raw Images Contains original Gel images. (PDF)

S2 File Contains Supplementary Tables S1-S17. (XLSX)

S3 File Contains Supplementary Figures S1-S10. (PDF)
