# Supplementary material for: Characterizing the circular RNA landscape in phloem sap of Brassica napus
Source: PLoS One. 2026 Apr 29;21(4):e0347473. doi: 10.1371/journal.pone.0347473 (PMC13127921; doi:10.1371/journal.pone.0347473)
Supplement: S1 Raw Images — (PDF) [file pone.0347473.s001.pdf]

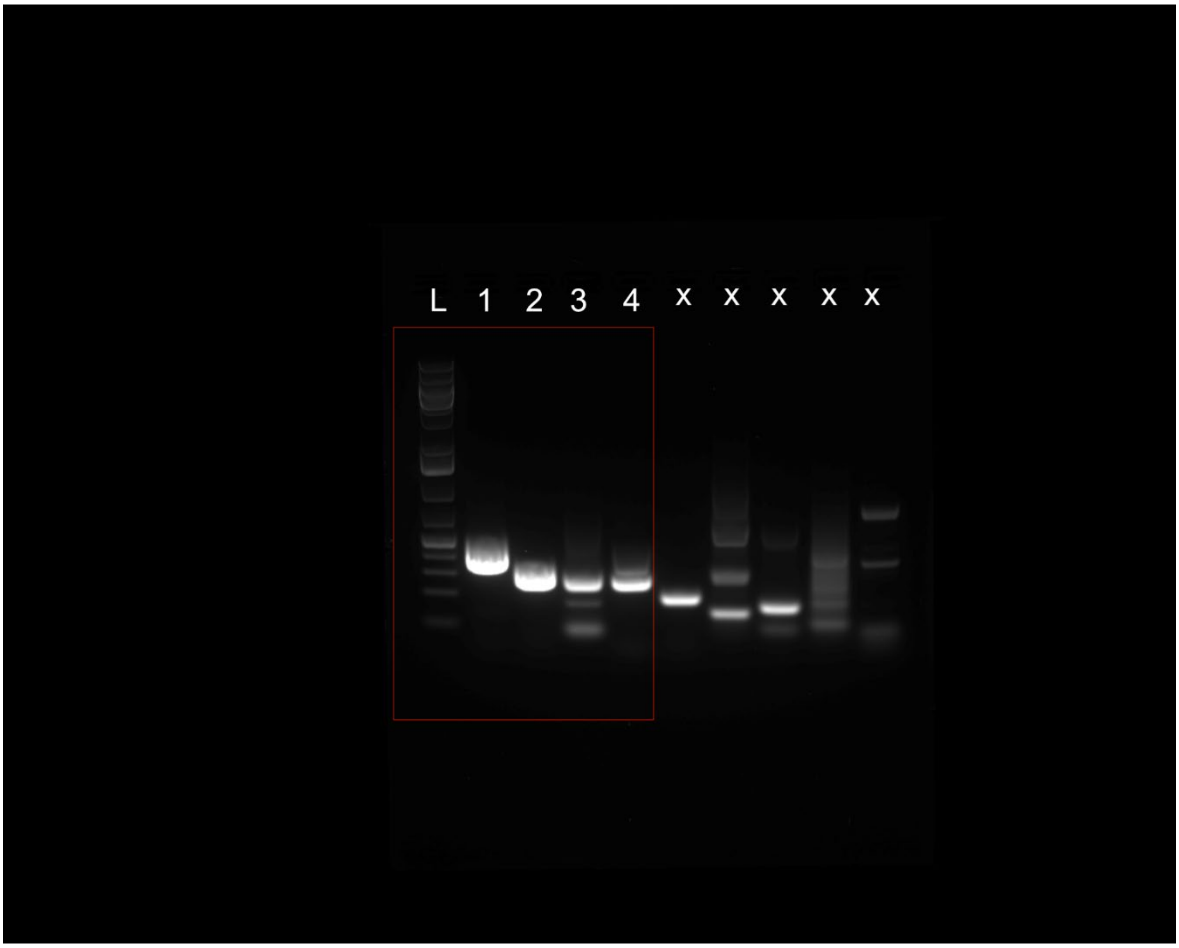

Figure 1: Original gel picture from Fig 2a. Area in red box is shown in Fig 2a, lanes marked with x are not included. L: 1 kb plus ladder from ThermoFisher, 1: circBnaC02g25110D(1,2,3,4), 2: circBnaEMB2423(5,6,7), 3: circBnaANL2(7,8), 4: circBnaInter05.

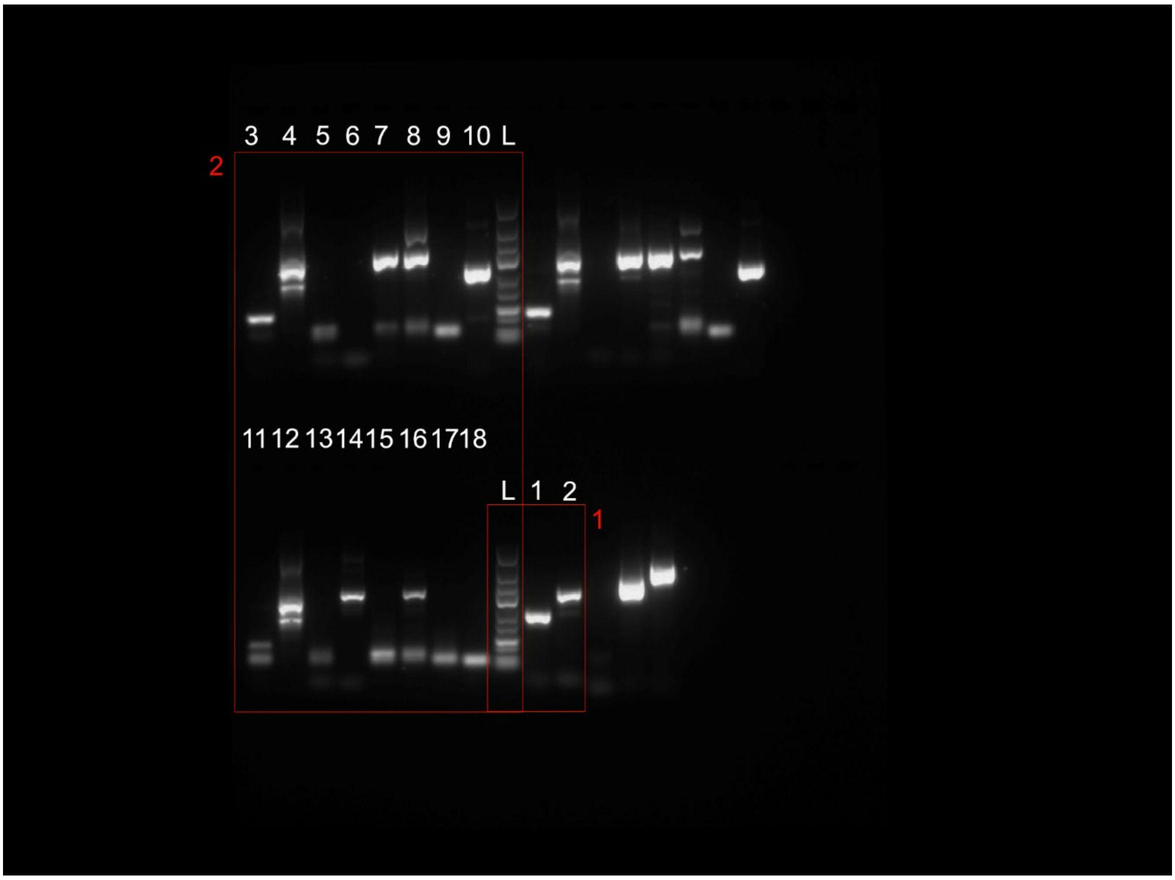

Figure 2: Original gel picture from Fig 2b (red box 1) and from S5b Fig (red box 2). Red boxes mark the area shown in Fig 2b and S5b Fig. L: 1 kb plus ladder from ThermoFisher. 1: At3g48470 divergent primer, 2: At3g48470 convergent primer, 3: circNUCL1 divergent primer phloem, 4: circNUCL1 convergent primer phloem, 5: circBnaT15B3 divergent primer phloem, 6: circBnaT15B3 convergent primer phloem, 7: circBnaEMB2423 divergent primer phloem, 8: circBnaEMB2423 convergent primer phloem, 9: circBnaNIPL2 divergent primer phloem, 10: circBnaNIPL2 convergent primer phloem, 11: circNUCL1 divergent primer leaf, 12: circNUCL1 convergent primer leaf, 13: circBnaT15B3 divergent primer leaf, 14: circBnaT15B3 convergent primer leaf, 15: circBnaEMB2423 divergent primer leaf, 16: circBnaEMB2423 convergent primer leaf, 17: circBnaNIPL2 divergent primer leaf, 18: circBnaNIPL2 convergent primer leaf.

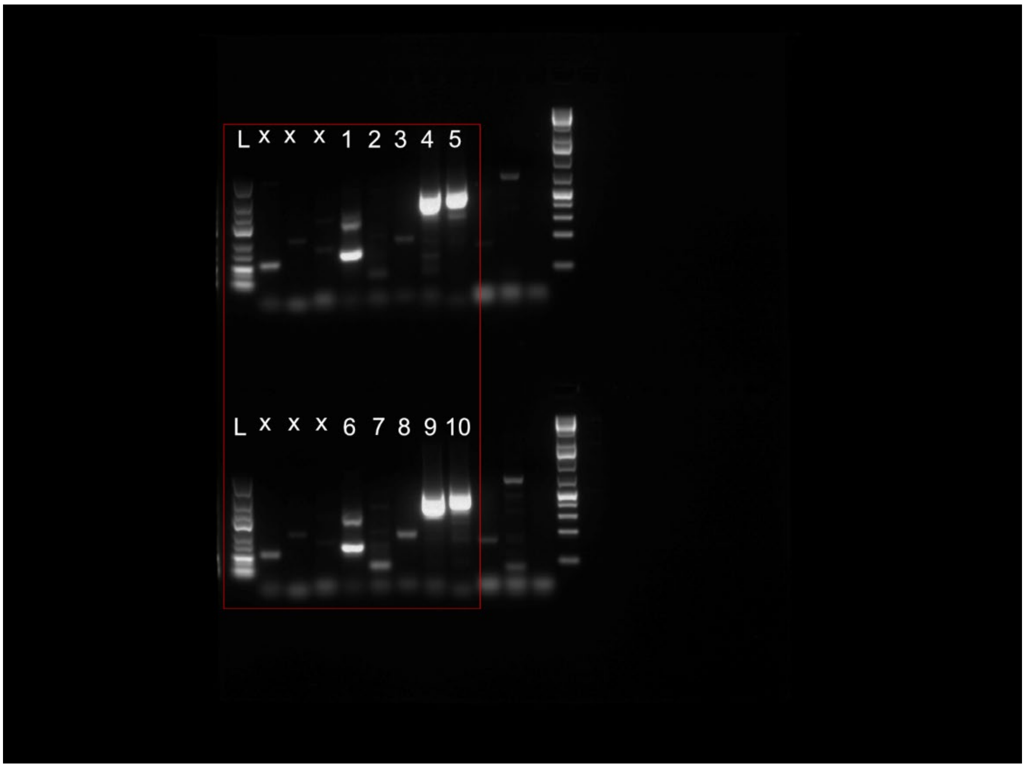

Figure 3: Original gel picture from S5a Fig. Red box marks the area shown in S5a Fig. X: lanes not included in S5a Fig, L: Low range DNA ladder ThermoFisher, 1: circBnaInter01 divergent primer phloem, 2: circBnaInter02 divergent primer phloem, 3: circBnaInter02 convergent primer phloem, 4: circBnaC02g25110D divergent primer phloem, 5: circBnaC02g25110D convergent primer phloem, 6: circBnaInter01 divergent primer leaf, 7: circBnaInter02 divergent primer leaf, 8: circBnaInter02 convergent primer leaf, 9: circBnaC02g25110D divergent primer leaf, 10: circBnaC02g25110D convergent primer leaf

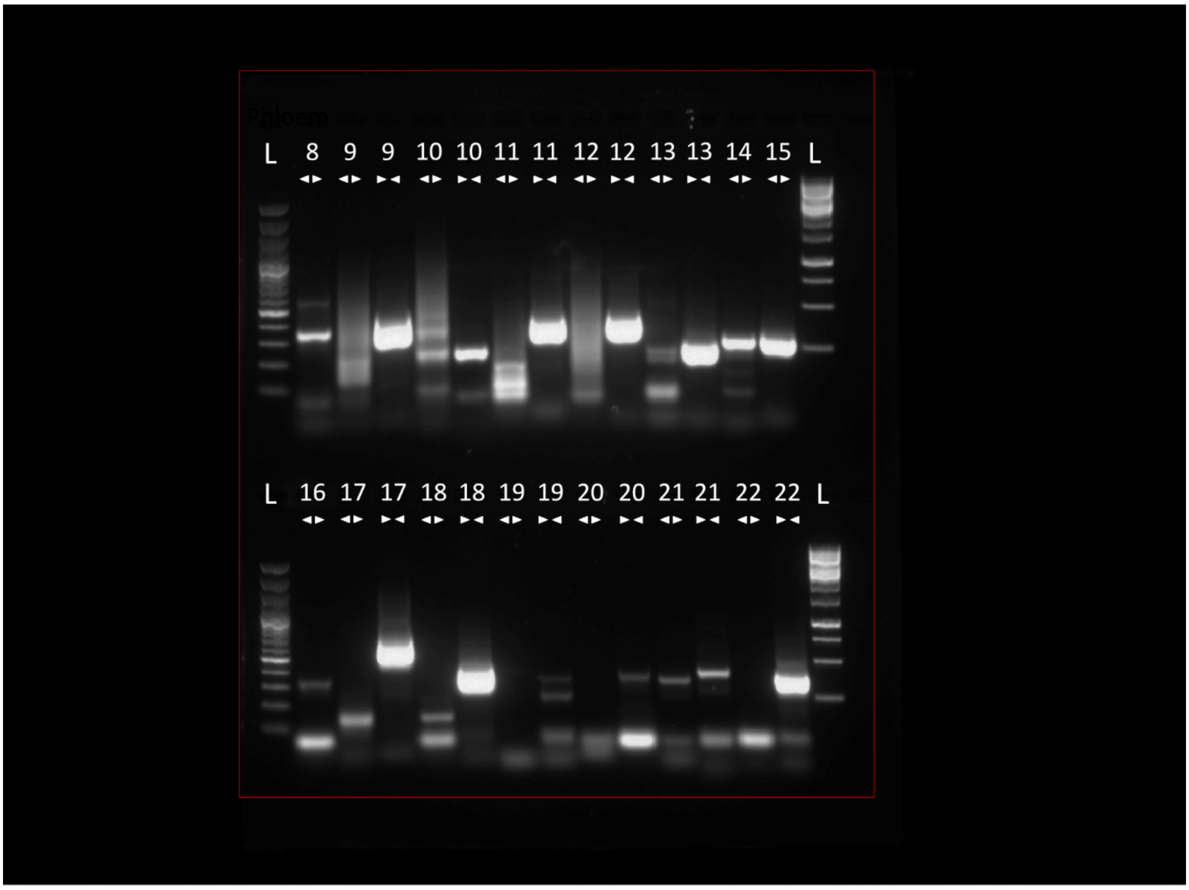

◀▶ divergent primer pair

▶◀ convergent primer pair

8: LK032104:439449-440239, circBnaInter04
9: LK032116:502414-502836, circBnaRBL
10: LK031847:562362-562748, circBnaA09g21600
11:LK031800:1071677-1072350, circBnaSYNC2
12: LK032116:501707-502081, circBnaRBL
13: LK031797:1502484-1502992, circBnaSUS
14: LK031889:298418-299059, circBnaInter05
15: LK032075:616214-617194, circBnaInter06
16: LK031822:1193072-1193636, circBnaInter07
17: LK032402:24013-24653, circBnaA05g33710D
18: LK031967:7043-7475, circBnaA09g38170D
19: LK031850:452816-454048, circBnaA07g34720D
20: LK032225:166944-168146, circBnaBCA1
21: LK032970:16048-16675, circBnaANL2
22:LK031789:187717-188328, circBnaC01g08350D

Figure 4: Original gel picture S5c Fig phloem. Red box marks the area shown in S5c Fig phloem.

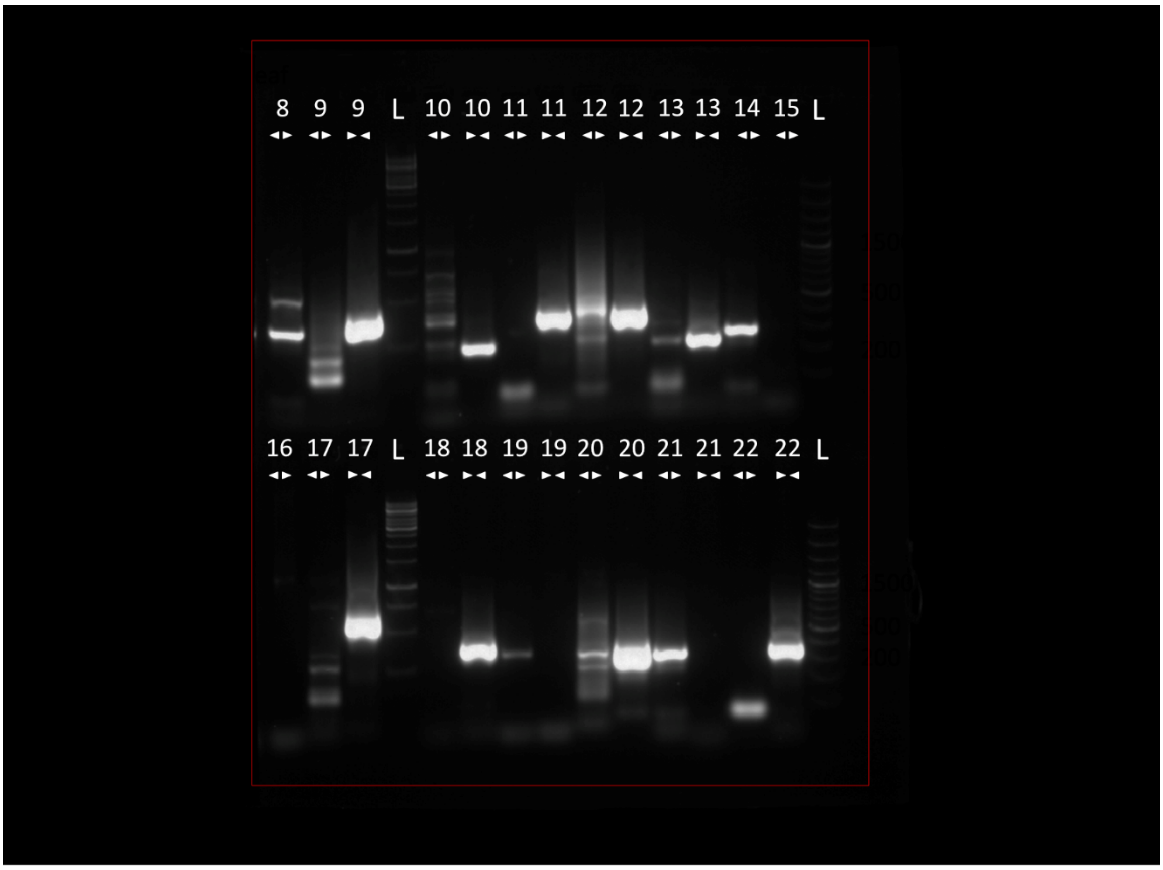

◀▶ divergent primer pair

▶◀ convergent primer pair

8: LK032104:439449-440239, circBnaInter04
9: LK032116:502414-502836, circBnaRBL
10: LK031847:562362-562748, circBnaA09g21600
11:LK031800:1071677-1072350, circBnaSYNC2
12: LK032116:501707-502081, circBnaRBL
13: LK031797:1502484-1502992, circBnaSUS
14: LK031889:298418-299059, circBnaInter05
15: LK032075:616214-617194, circBnaInter06
16: LK031822:1193072-1193636, circBnaInter07
17: LK032402:24013-24653, circBnaA05g33710D
18: LK031967:7043-7475, circBnaA09g38170D
19: LK031850:452816-454048, circBnaA07g34720D
20: LK032225:166944-168146, circBnaBCA1
21: LK032970:16048-16675, circBnaANL2
22:LK031789:187717-188328, circBnaC01g08350D

Figure 5: Original gel picture S5c Fig leaf. Red box marks the area shown in S5c leaf. L: 1kb plus ladder from ThermoFisher or Low Range DNA ladder from ThermoFisher.

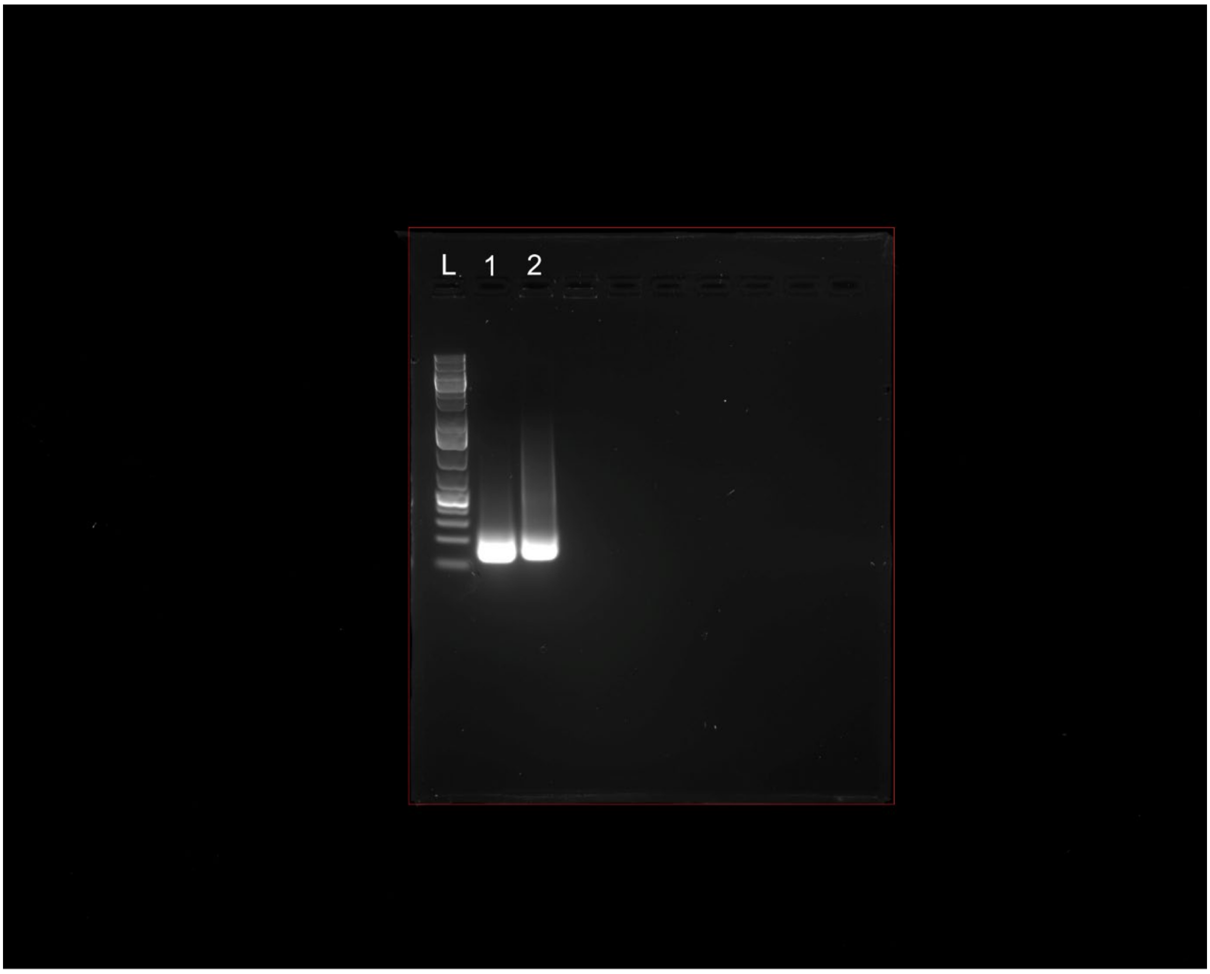

Figure 6: Original gel picture S8 Fig. Red box marks the area shown in S8. L: 1kb plus ladder from ThermoFisher, 1: circANL2 sample 1 in vitro circularized RT-PCR with divergent primer, 2: circANL2 sample 2 in vitro circularized RT-PCR with divergent primer

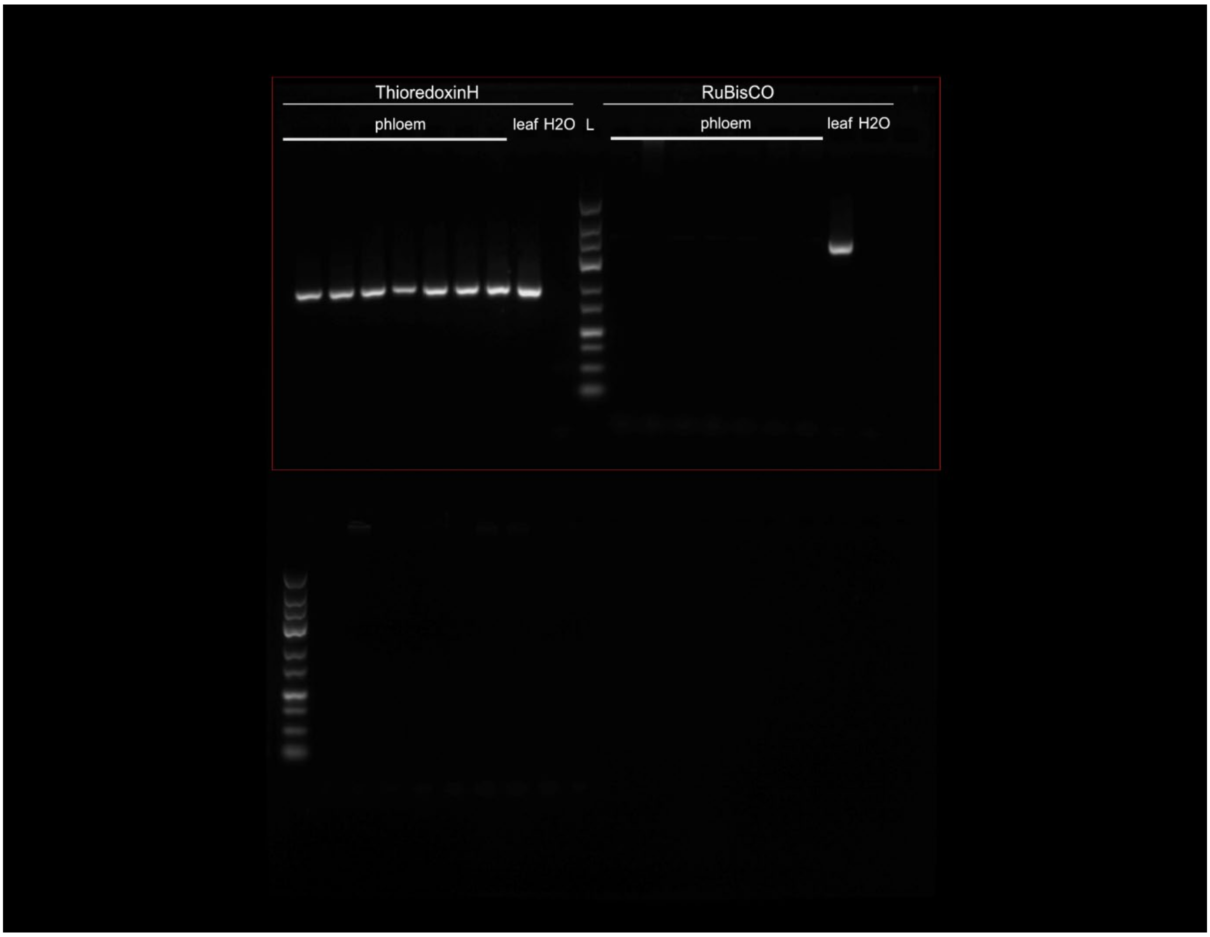

Figure 7: Original gel picture from S9 Fig. Red box marks area shown in S9 Fig. L: 1kb plus ladder from ThermoFisher, Primers targeting ThioredoxinH or RuBisCO were used in RT-PCR on multiple phloem samples and one leaf sample as control to test the purity of the phloem.
