# Supplementary material for: Characterizing the circular RNA landscape in phloem sap of Brassica napus
Source: PLoS One. 2026 Apr 29;21(4):e0347473. doi: 10.1371/journal.pone.0347473 (PMC13127921; doi:10.1371/journal.pone.0347473)
Supplement: S3 File — (PDF) [file pone.0347473.s003.pdf]

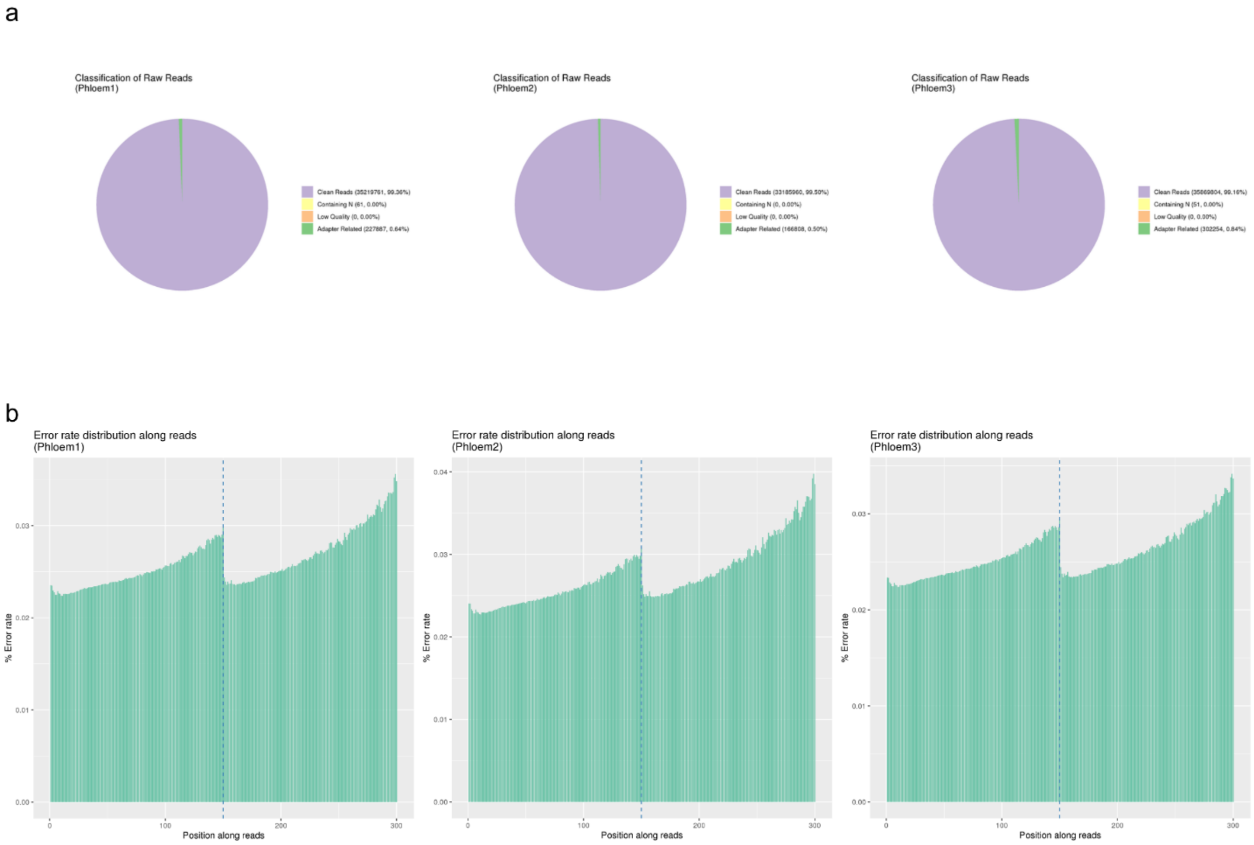

S1 Fig: Quality control of all three replicates of the rRNA-depleted library. a) Classification of raw reads. b) Error rate distribution along the reads.

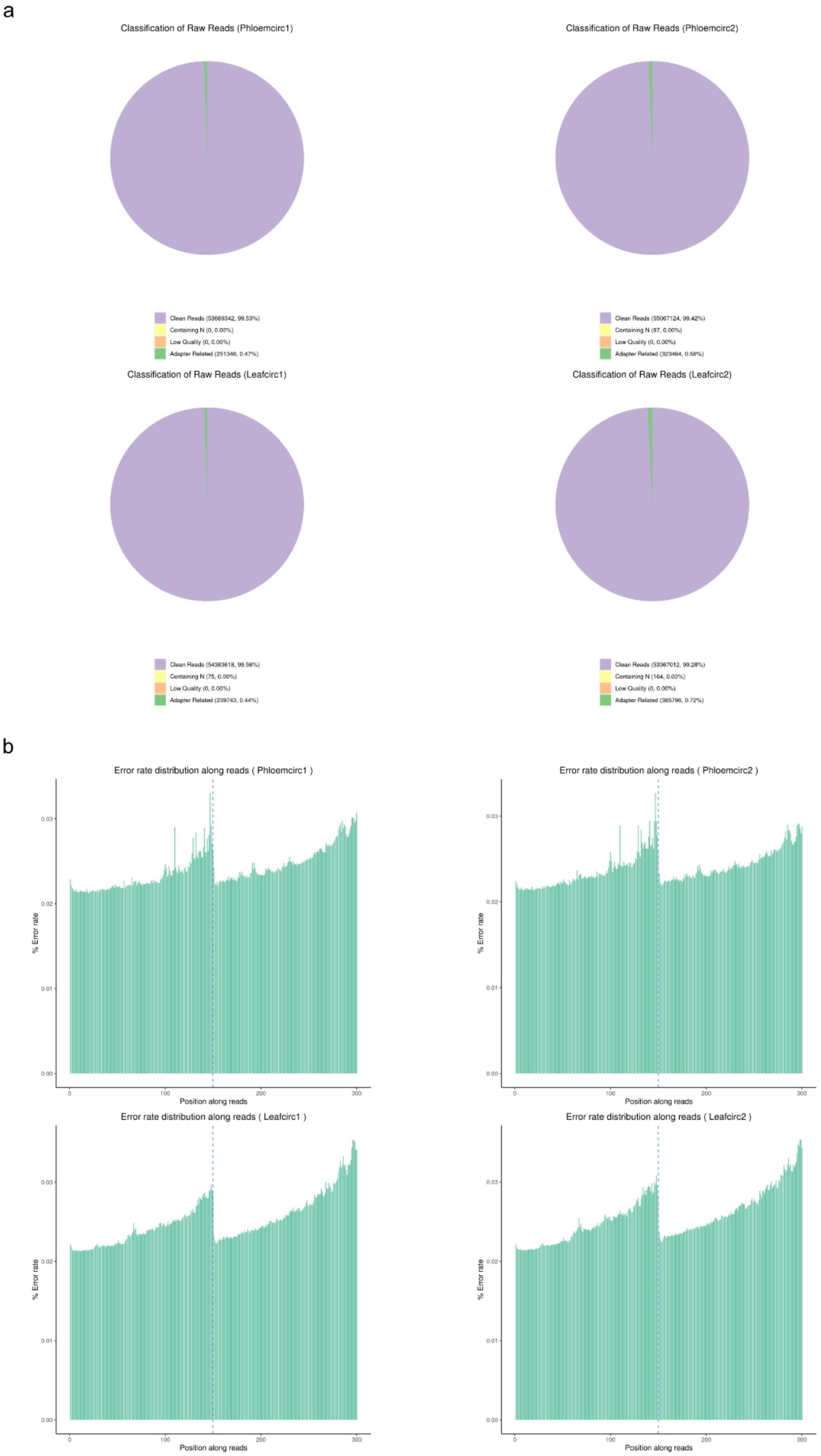

S2 Fig: Quality control of phloem and leaf replicates of the circRNA-enriched libraries. a) Classification of raw reads. b) Error rate distribution along reads.

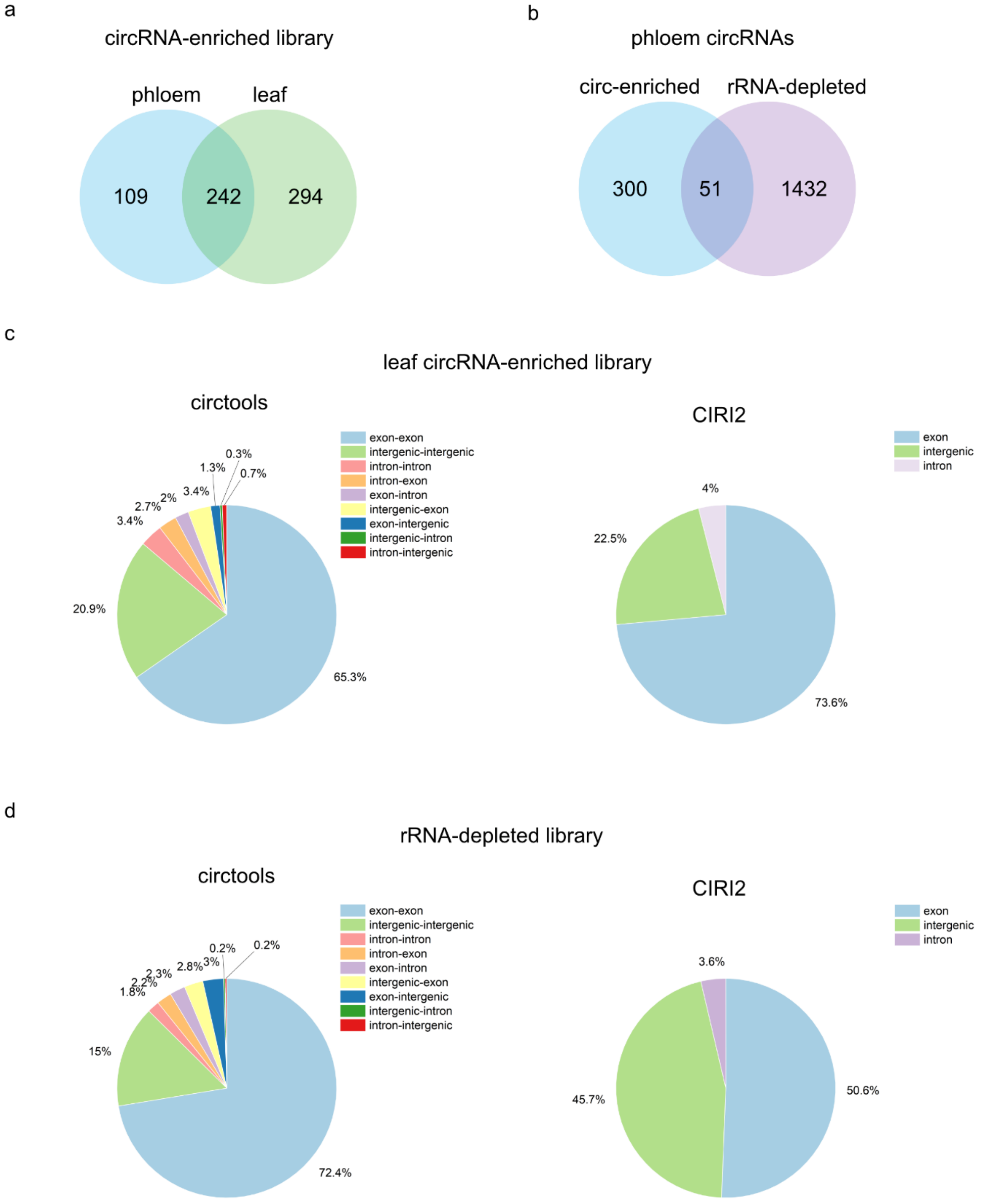

S3 Fig: Detected circRNAs in circ-enriched and lnc-libraries from phloem RNAs. a) Venn-diagram of circRNAs from the circRNA-enriched library compared to circRNAs from the rRNA-depleted library identified by circtools and CIRI2. **b)** Venn-diagram of circRNAs identified in phloem and in leaf samples from circRNA-enriched libraries with circtools and CIRI2. **c)** Origin of circRNAs in the circRNA-enriched library of leaf samples identified by circtools and CIRI2. **d)** Origin of circRNAs in phloem samples of the rRNA-depleted library identified by circtools and CIRI2.

a

b

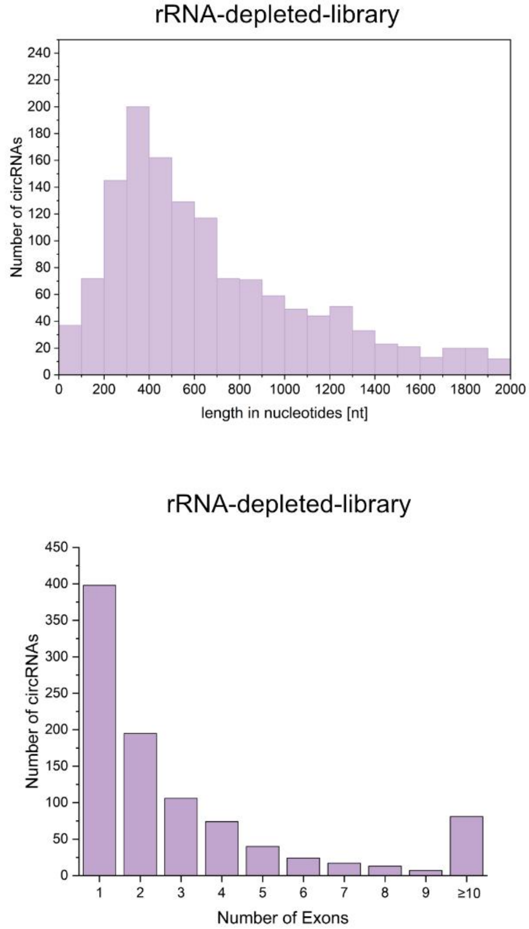

S4 Fig: Length distribution and exon number of circRNAs from lnc-library. a) length distribution of circRNAs from the rRNA-depleted library The x-axis displays the length in nucleotides, and the y-axis displays the number of circRNAs. **b)** The number of circRNAs spanning a certain number of exons from the rRNA-depleted library. The x-axis displays the number of exons and the y-axis the number of circRNAs.

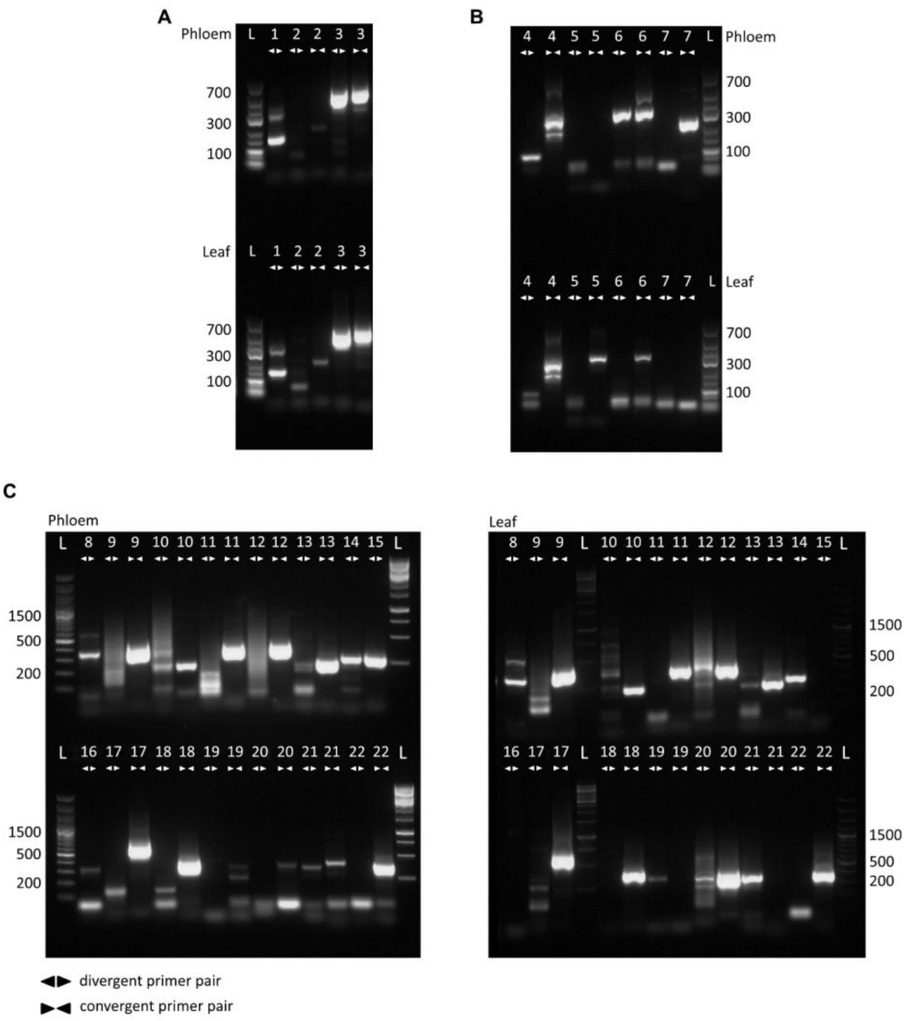

1: LK031823:1895973-1903835, circBnaInter01
2: LK032206:21055-21587, circBnaInter02
3: LK032045:141379-143249, circBnaC02g25110D
4: LK034680:3566-3646, circNUCL1
5: LK032960:6840-6928, circBnaT15B3_160()
6: LK031813:2017074-2018095, circBnaEMB2423(5,6,7)
7: LK031797:1982759-1983857, circBnaNIPL2(1,2,3,4)
8: LK032104:439449-440239, circBnaInter04
9: LK032116:502414-502836, circBnaRBL
10: LK031847:562362-562748, circBnaA09g21600
11:LK031800:1071677-1072350, circBnaSYNC2
12: LK032116:501707-502081, circBnaRBL
13: LK031797:1502484-1502992, circBnaSUS
14: LK031889:298418-299059, circBnaInter05
15: LK032075:616214-617194, circBnaInter06
16: LK031822:1193072-1193636, circBnaInter07
17: LK032402:24013-24653, circBnaA05g33710D
18: LK031967:7043-7475, circBnaA09g38170D
19: LK031850:452816-454048, circBnaA07g34720D
20: LK032225:166944-168146, circBnaBCA1
21: LK032970:16048-16675, circBnaANL2
22:LK031789:187717-188328, circBnaC01g08350D

S5 Fig: Agarose gels of amplicons from divergent and convergent primer pairs targeting predicted circRNAs. Ladder: 1 kb plus DNA ladder (ThermoFisher Scientific) and Low Range DNA ladder (ThermoFisher Scientific).

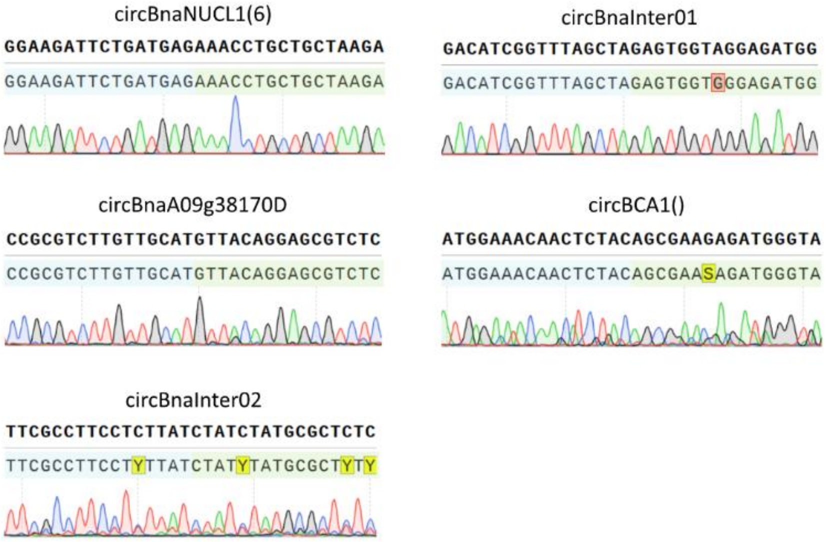

S6 Fig: Sanger sequencing of BSJ-amplicons. Sanger sequencing results of BSJ amplicon sequencing for circRNAs circBnaNUCL1(6), circBnaInter01, circBnaA09g38170D, circBCA1() and circBnaInter02.

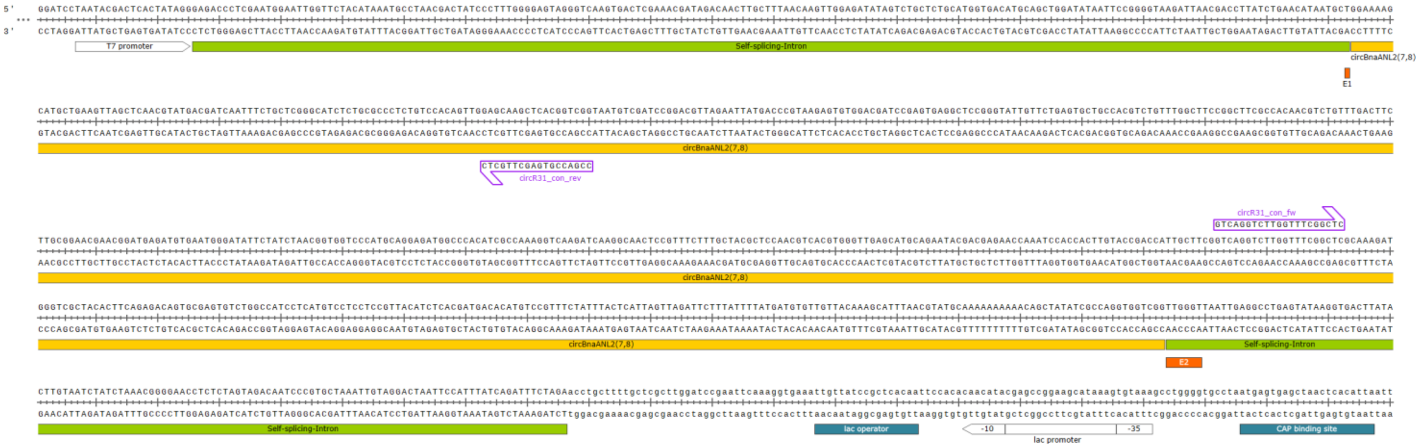

S7: Self-splicing Intron construct for in vitro circularization of circBnaANL2.

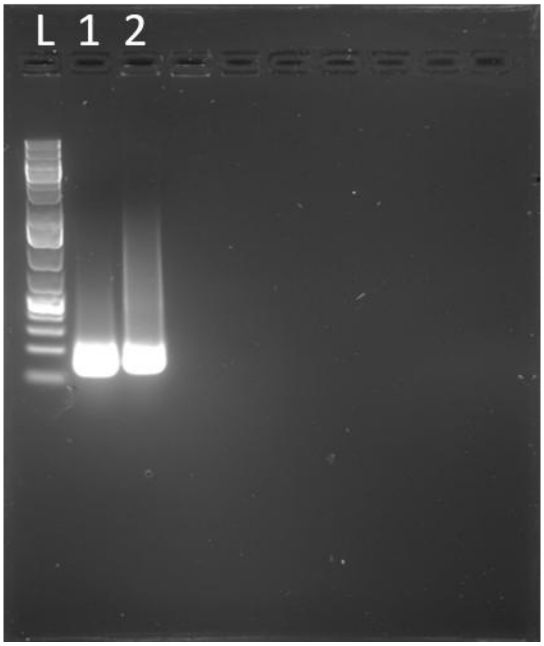

S8 Fig: Amplicons from PCR with divergent primers after in vitro RNA circularization, RNaseR digestion and cDNA synthesis. L: Ladder, 1kb Ladder Thermo Fisher Scientific; 1: Sample 1; 2: Sample 2. 1.5 % agarose gel.

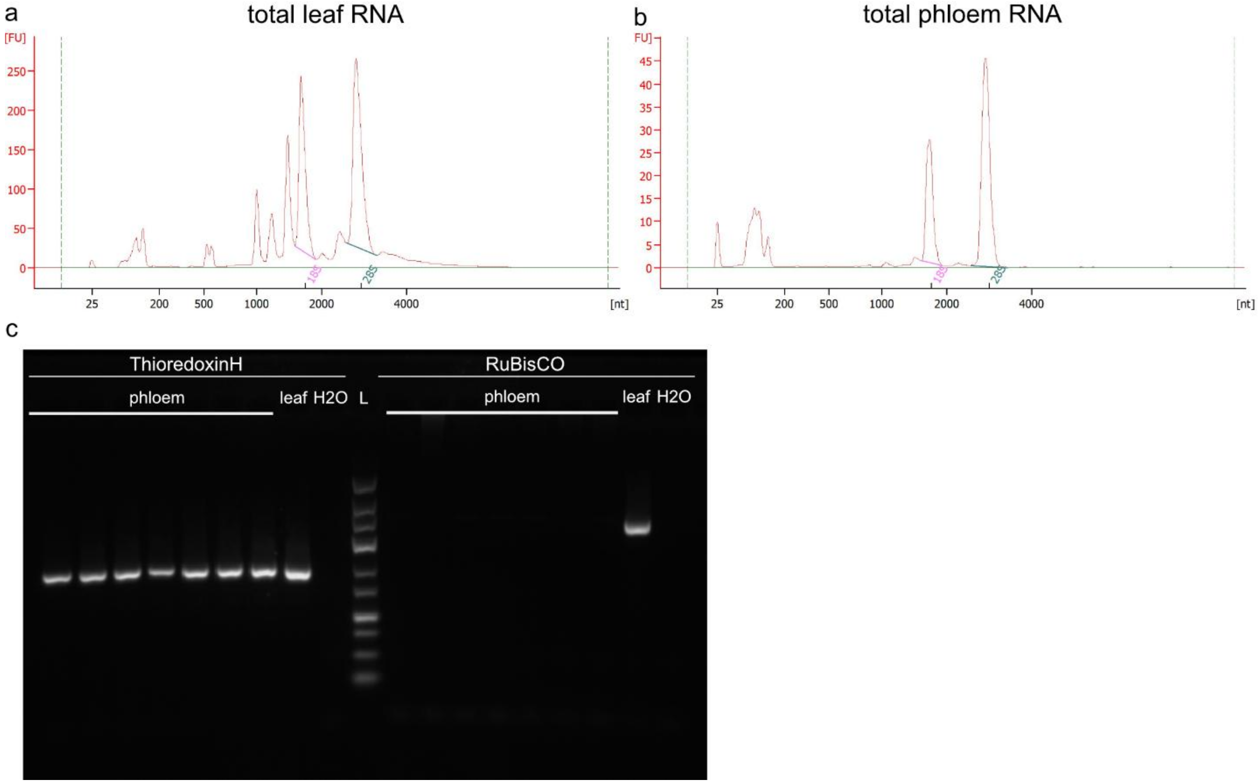

S9 Fig: Phloem purity shown with Bioanalyzer graphs and RT-PCR. a) Bioanalyzer graph from total leaf RNA. b) Bioanalyzer graph from total phloem RNA. c) Agarose gel with amplicons from RT-PCR with primers targeting ThioredoxinH or RuBisCO. Multiple biological replicates from total phloem RNA and one from leaves were tested. L: 1kb plus ladder from ThermoFisher.

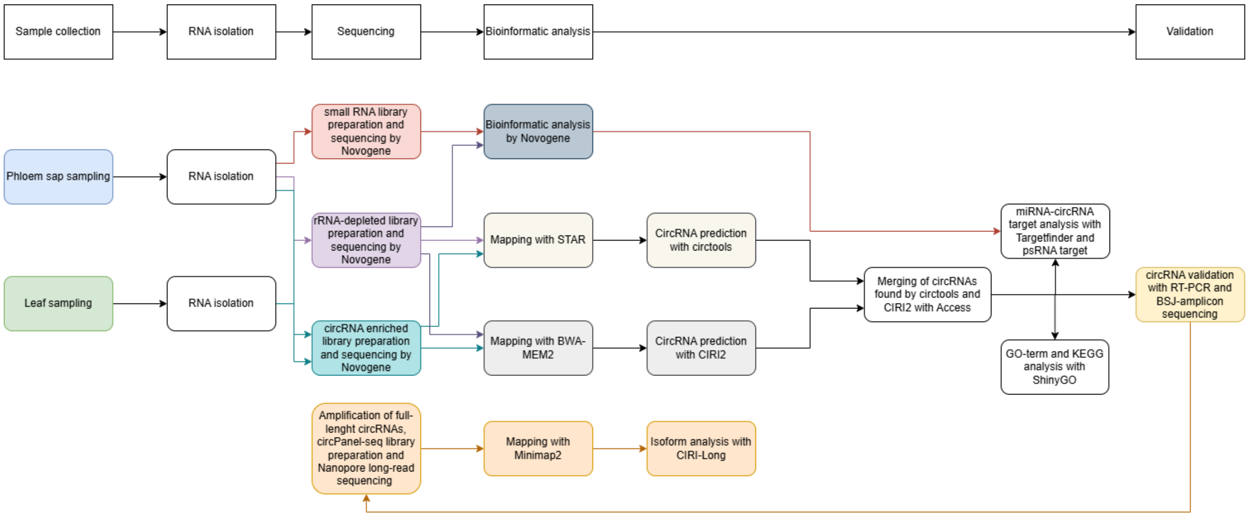

S10 Fig: Bioinformatic workflow for circRNA identification and downstream analysis.
